# Multiplex, Real-Time, Point-of-care RT-LAMP for SARS-CoV-2 Detection Using the HFman Probe

**DOI:** 10.1021/acssensors.1c02079

**Published:** 2022-02-22

**Authors:** Yajuan Dong, Yongjuan Zhao, Shenwei Li, Zhenzhou Wan, Renfei Lu, Xianguang Yang, Guoying Yu, Julien Reboud, Jonathan M. Cooper, Zhengan Tian, Chiyu Zhang

**Affiliations:** † College of Life Sciences, Henan Normal University, Xinxiang 453007, China; ‡ Shanghai Public Health Clinical Center, Fudan University, Shanghai 201508, China; § Shanghai International Travel Healthcare Center, Shanghai 200335, China; ∥ Medical Laboratory of Taizhou Fourth People’s Hospital, Taizhou 225300, China; ⊥ Clinical Laboratory, Nantong Third Hospital Affiliated to Nantong University, Nantong 226006, China; # Division of Biomedical Engineering, University of Glasgow, G12 8LT Glasgow, U.K.

**Keywords:** multiplex LAMP, HFman probe, high-fidelity DNA polymerase, non-specific amplification, COVID-19/SARS-CoV-2, point-of-care testing (POCT)

## Abstract

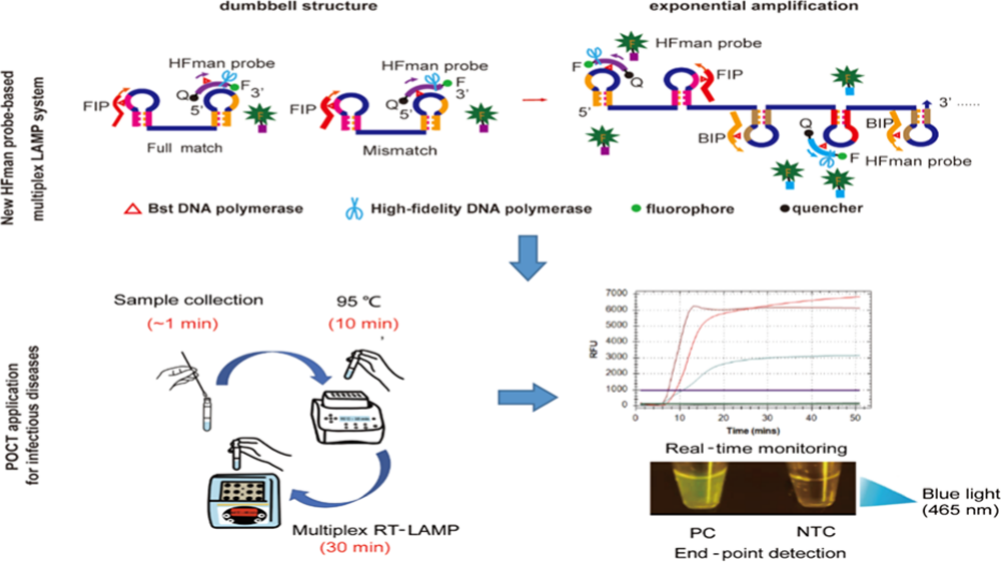

Viral evolution impacts diagnostic test performance through the emergence of variants with sequences affecting the efficiency of primer binding. Such variants that evade detection by nucleic acid-based tests are subject to selective pressure, enabling them to spread more efficiently. Here, we report a variant-tolerant diagnostic test for SARS-CoV-2 using a loop-mediated isothermal nucleic acid-based amplification (LAMP) assay containing high-fidelity DNA polymerase and a high-fidelity DNA polymerase-medicated probe (HFman probe). In addition to demonstrating a high tolerance to variable SARS-CoV-2 viral sequences, the mechanism also overcomes frequently observed limitations of LAMP assays arising from non-specific amplification within multiplexed reactions performed in a single “pot”. Results showed excellent clinical performance (sensitivity 94.5%, specificity 100%, *n* = 190) when compared directly to a commercial gold standard reverse transcription quantitative polymerase chain reaction assay for the extracted RNA from nasopharyngeal samples and the capability of detecting a wide range of sequences containing at least alpha and delta variants. To further validate the test with no sample processing, directly from nasopharyngeal swabs, we also detected SARS-CoV-2 in positive clinical samples (*n* = 49), opening up the possibility for the assay’s use in decentralized testing.

## Introduction

The coronavirus pandemic, caused by SARS-CoV-2, has resulted in over 272 million infections and over 5.3 million deaths (14 December 2021). Its spread globally can be attributed both to the fact that asymptomatic and pre-symptomatic individuals are infectious,^1,2^ as well as to the emergence of variants.^3^ Currently, genotyping of the SARS-CoV-2 is enabling near “real-time” information on the genetic sequences of the circulating virus, with mutation rates of ∼2 nucleotides/month, putting at risk diagnostic detection strategies that do not accommodate changes in the viral genome,^4^ once sequencing efforts decrease, or for other highly variable viruses. In order to contain the transmission of SARS-CoV-2, we propose that rapid and sensitive nucleic acid amplification (NAA) and detection methods are necessary, which are not only tolerant to the evolution of variants but are also simple to perform, requiring only minimal sample preparation.

NAA tests (NAATs) have been widely used for the clinical detection of infectious pathogens due to their high sensitivity and specificity.^5,6^ The current gold standard method remains the reverse transcription quantitative polymerase chain reaction (RT-qPCR).^7,8^ However, it is time consuming and requires precise thermal cycling, limiting its application in decentralized situations and especially in resource-limited settings (as PCR requires advanced equipment with highly trained personnel needed for sample processing). As an alternative, isothermal NAA, including loop-mediated isothermal amplification (LAMP), has been used in point-of-care testing for infectious diseases.^5,9−13^

To date, only a few LAMP assays have been approved for clinical application^14^ due in part to their low tolerance to highly variable target sequences, frequent non-specific amplification,^15,16^ and the limitations associated with multiplexing in a single reaction, which is challenging to achieve^17^ at high sensitivities (e.g., <10^4^ copies per milliliter)^18^ without complex molecular designs.^19−21^

We previously developed a mismatch-tolerant LAMP method that improves the amplification efficiency of highly variable target sequences for human RNA viruses such as HIV-1,^22^ Dengue virus,^23^ and SARS-CoV-2.^24,25^ In this study, we demonstrate a multiplex LAMP system that uses a high-fidelity DNA polymerase-mediated fluorescent probe (HFman probe) to improve specificity and, importantly, in contrast to other amplification strategies (e.g., SHERLOCK^4^), allows us to realize single-pot multiplex detection without the need for RNA extraction (sample processing) or indeed a separate reverse transcription (RT) step.

**Figure 1:**
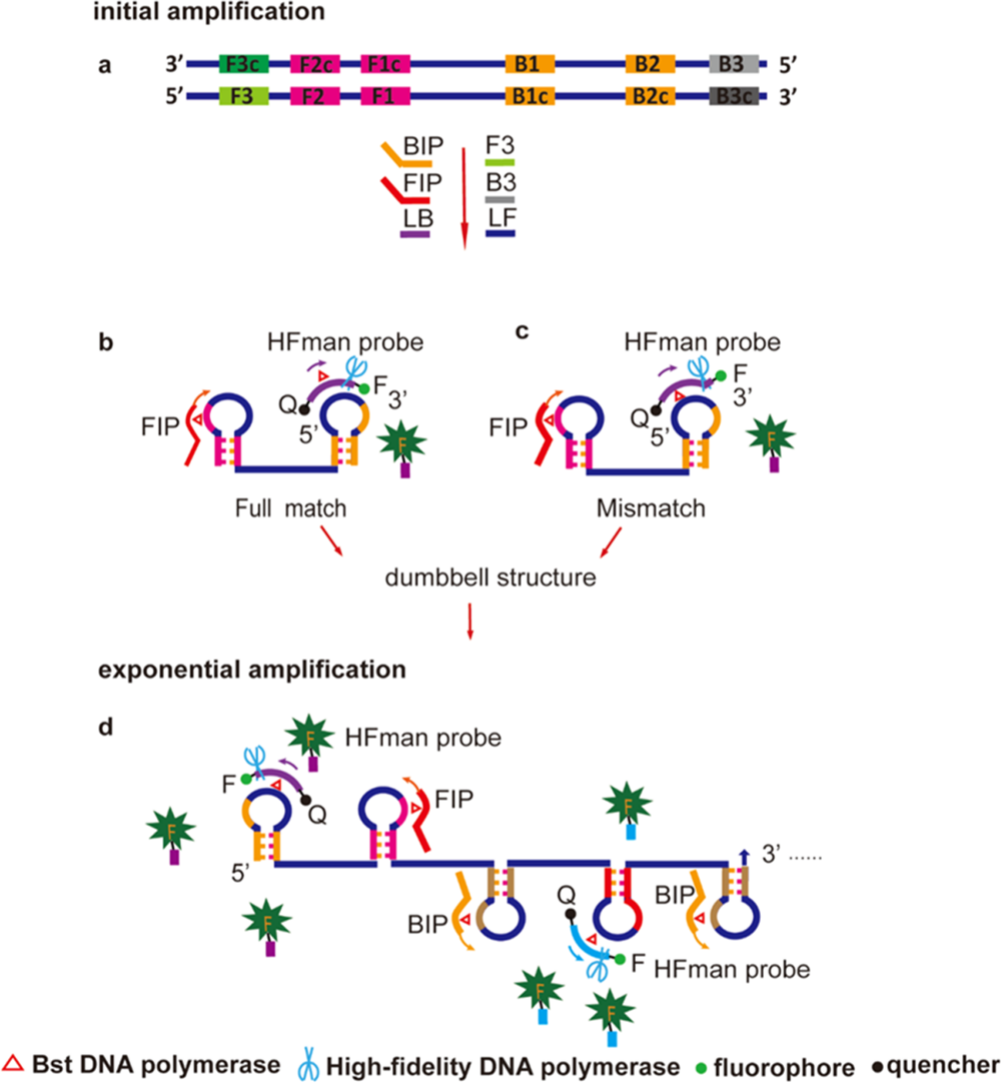
Principle of the multiplex real-time RT-LAMP. The specific fluorescent signal of the multiplex real-time LAMP is mediated by a small amount of high-fidelity DNA polymerase with the HFman probe. For simplicity of illustration, only one probe molecule is shown in this figure, but other fluorochromes (e.g., FAM, CY5, and HEX) with different fluorescence wavelengths can be used to label different probes for different targets in a multiplexed format. During the initial LAMP phase, the primers bind to the target sequence to start the LAMP process (a). After a dumbbell structure is formed, its loop regions provide binding sites for the HFman probe that has the same sequence as the loop primer (LF or LB) (b,c). The HFman probe is recognized and cleaved by high-fidelity DNA polymerase when it specifically hybridizes to the loop region without (b) or with a mismatch with the loop region (c) to release the fluorescent signal and to expose free 3′-OH for further extension by Bst DNA polymerase. (d) Hybridized FIP/BIP and LF/LB/HFman probes initiate DNA extension by Bst DNA polymerase. Newly synthesized DNA strands form dumbbell structures to start self-priming extension. During the extension, the fluorescence signal increases exponentially as the fluorophore is released from its quenching pair in the HFman probe.

In our assay (Figure 1), the fluorescent probe comprises an oligonucleotide labeled by a fluorophore and a quencher at the 3′ and 5′ ends, respectively. As the probe is cleaved by high-fidelity DNA polymerase, releasing a fluorescent signal,^26^ the mechanism is named after the high-fidelity DNA polymerase-mediated probe (HFman probe). The assay not only targets the open reading frame (ORF) and E genes of SARS-CoV-2 but also incorporates a human housekeeping gene, β-actin, requiring triplex detection all within a single pot. Clinical validation, with only minimal sample manipulation or processing when assayed directly from a nasopharyngeal swab, demonstrated that our SARS-CoV-2 multiplex RT-LAMP assay had very good sensitivity and analytical specificity compared with a commercial RT-qPCR assay as the gold standard. The microbial specificity of the assay was also confirmed by using a panel of 17 common respiratory viruses, including HCoV-HKU-1; HCoV-NL63; HCoV-OC43; HCoV-229E; influenza A, B, and C; parainfluenza type 1–3; enterovirus; RSV A and B groups; human rhinovirus; human metapneumovirus; adenovirus; and bocavirus, with no amplification signal observed.

## Results

### Principle of the Real-Time Multiplex LAMP

Our multiplex real-time LAMP assay uses a small amount of high-fidelity DNA polymerase and an HFman probe in a standard LAMP reaction system and is illustrated in the generalized scheme shown in Figure 1. The HFman probe has the same sequence as a loop primer LF or LB (Figure 1b–d) and forms a dumbbell-shaped secondary structure in which a fluorophore signal remains attenuated by a quencher.

During the initial amplification phase, the primers bind to the target sequence to initiate DNA extension (Figure 1a). As the dumbbell structure is generated, the loop regions of the dumbbell structure provide binding sites for either the loop primers or the HFman probes, depending on the concentrations and the stoichiometry of the reaction. When hybridized, the 3′ fluorophore and/or 3′ fluorescence-labeled base of the HFman probe, regardless of any mismatches with the loop regions of the dumbbell structures (Figure 1b,c), are recognized as a damaged base and excised by the high-fidelity DNA polymerase added to the reaction mix, releasing the quenched fluorescent signal (Figure 1d).

At the same time, excision exposes the free 3′-hydroxyl group (−OH) of the probe, which enables the probe to act as a primer to initiate DNA extension by Bst DNA polymerase (Figure 1d). As the LAMP reaction progresses, the fluorescence signal increases exponentially as the fluorophore is released from its quenching pair (Figure 1d).

Not only can the reaction be monitored simply by measuring fluorescence intensity, but multiplexing can also be readily implemented using different fluorescent groups on HFman probes of different sequences.

### Real-Time Multiplex RT-LAMP Assay

To demonstrate that high-fidelity DNA polymerase mediates the real-time monitoring of RT-LAMP, we designed an HFman probe targeting the ORF gene of SARS-CoV-2. Figure S1a shows an amplification curve for the reaction with target RNA, while no amplification signal was detected in the reaction without the template, indicating that high-fidelity DNA polymerase recognizes and cleaves the HFman probe that specifically hybridized with the template, therefore mediating the real-time LAMP.

The mechanism of the assay is resilient to 3′ mismatches, as shown in Figure S1b. A mutant HFman probe containing a mismatched 3′-terminal base (A → G) with target RNA, generated similar amplification curves, indicating that the high-fidelity DNA polymerase can specifically cleave the probes that bind to the target sequence, regardless of the presence or absence of a mismatched base at the 3′ end.^26,27^ High-fidelity DNA polymerase not only cleaves the last 3′-5′ phosphodiester bond of the probe that links the fluorescence group, but also the 3′ second last phosphodiester bond that links the last nucleotide.

To establish the potential of the HFman probe strategy to mediate a multiplexed assay, we further studied the cleavage site of high-fidelity DNA polymerase in the probe. We designed a series of specific HFman probe constructs (as the LB primer) targeting the E gene of SARS-CoV-2 (Figure S1c). These used a BHQ1 group at the 5′ end, together with a FAM fluorophore at the 3′-OH, or a HEX label on the 3′ last base T, the latter having either a free 3′-OH (3′-free-T-probe) or a C3 spacer to block the 3′-OH (3′-blocked-T-probe). All three probes generated amplification curves, including the blocked probe, as only one of the primers is blocked, while all others can mediate the amplification. However, the reactions with the 3′-blocked-T-probe and the HFman probe labeled by FAM at 3′-OH were slower than with the 3′-free-T-probe (Figure S1d).

We propose that high-fidelity DNA polymerase will only cleave the final phosphodiester bond linking the terminal nucleotide with free-3′-OH, although it will cleave the last two phosphodiester bonds when 3′-OH is blocked, one linking fluorescence or other chemical groups and another linking the last nucleotide (Figure S1c). This allows us to design a multiplex assay using the cleavage site to specifically release the fluorescence signal.

### Optimization of Real-Time Multiplex RT-LAMP

We have previously observed that 0.15 U of high-fidelity DNA polymerase in a 25 μL reaction is the optimal concentration for carrying out the 3′-5′exonuclease activity while not interfering with Bst or Taq DNA polymerase for primer extension.^23,27^ In this instance, reactions between 0.1 and 0.5 U Q5 with high-fidelity DNA polymerase showed similar speeds (Figure S2a), although higher fluorescence intensities were observed in reactions with 0.1 and 0.2 U Q5 DNA polymerase. Consequently, a concentration of 0.15 U of Q5 DNA polymerase per 25 μL reaction was selected for subsequent experiments. All reactions with HFman probe concentrations from 0.1 to 0.4 μM generated similar time threshold (Tt) values (Figure S2b), although the strongest fluorescence intensity was observed for 0.4 μM. The HFman probe sequence was the same as that of the FL primer, serving as a primer in the reaction only when the 3′ blocked groups were removed by high-fidelity DNA polymerase, such that increasing the FL primer concentrations has the potential to further improve performance. Results show that using an equal proportion of the HFman probe and FL primer (0.2 μM each) generated optimal performance, and it was selected for subsequent experiments (Figure S3).

At least two different genes are recommended as the gold standard to confirm COVID-19 infection using RT-PCR.^28^ To establish a multiplex real-time RT-LAMP assay for SARS-CoV-2 detection, we successfully combined the primers and HFman probes specific for ORF and E genes of SARS-CoV-2, optimized the concentrations of the ORF and E primers and probes (Figure S4), and added the human β-actin gene as an internal control (Figure S1e).

### Tolerance of the Real-Time Multiplex RT-LAMP to Highly Variable Target Sequences

**Figure 2:**
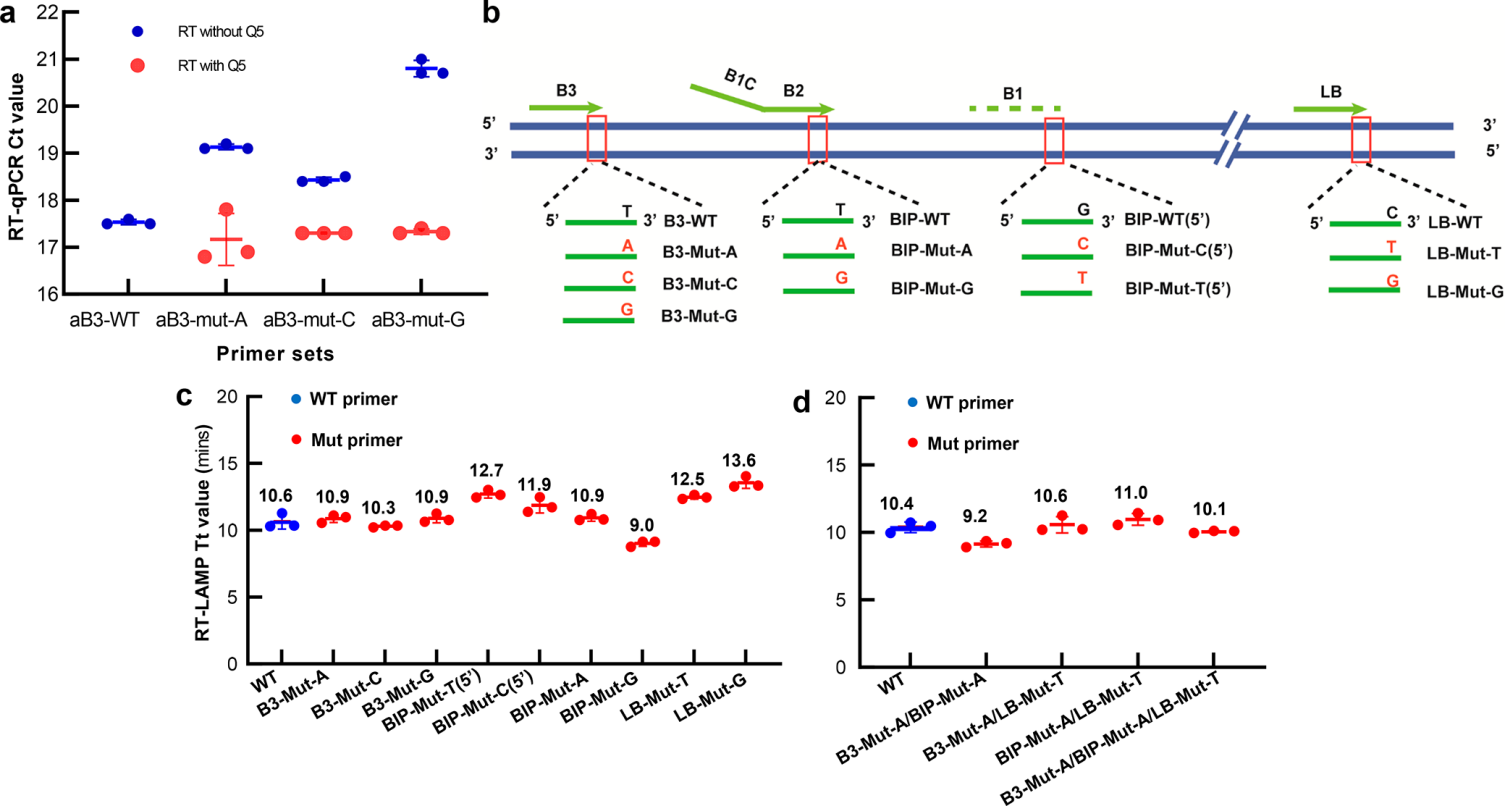
Influence of highly variable target sequences on amplification efficiency of the HFman-based real-time RT-LAMP. (a) Functional verification of high-fidelity DNA polymerase to cleave 3′ mismatches in RT reaction. The RT reactions were performed using wild-type or mutant B3 primers (Table S1) in two groups, namely with and without Q5 high-fidelity DNA polymerase (Figure S5). Ct values of the qPCR assay using different cDNA products from the RT reactions are shown. (b) Design and information of wild-type (WT) and mutant (Mut) primers. (c,d) Performance comparisons of the RT-LAMP assays using the primer set containing one mutant primer (c) and two to three concomitant different mutant primers (d) with the assay using the WT primer set. WT: wild-type and Mut: mutant. Q5: Q5 high-fidelity DNA polymerase. Tt: time threshold of the real-time RT-LAMP.

We tested whether the real-time multiplex RT-LAMP assay can detect highly variable targets as mismatched sequences. Although the RT step is part of the RT-LAMP sample preparation, to simplify this, we used the proof-reading activity of high-fidelity DNA polymerase in a 3′ mismatch of a DNA–RNA duplex (Figure 2a). The BIP and B3 primers bind to RNA and extend to form cDNA.

We designed a B3 primer with an adaptor at its 5′ end (known as aB3-WT) and three mutant aB3 primers with the other bases at their 3′-ends (known as aB3-Mut-A/C/G) (sequences provided in Table S1). We performed RT reactions with and without additional high-fidelity DNA polymerase using aB3-WT and its three mutant primers. The obtained cDNA was subjected to a subsequent qPCR assay using the F3 primer and the adaptor (see Figure S5a, graphical representation and the Supporting note method for details).

Without high-fidelity DNA polymerase, the three aB3-Mut primers generated substantially higher Ct values (about 0.9–3.2 higher Ct) than the aB3-WT primer (Figure 2a), implying that about 1.9–9.4 times less cDNA was generated by aB3-Mut primers than by the aB3-WT primer in the standard RT reaction. This result indicates that in spite of the fact that the RT enzyme is an error-prone polymerase and can extend from a mismatched base, mismatches at the 3′-end of the primer still reduced cDNA synthesis efficiency. When the high-fidelity DNA polymerase was added to the RT reaction, the obtained cDNA generated substantially lower Ct values (about 1.1–3.4 lower Ct) for three aB3-Mut primers than the cDNA from RT reaction without high-fidelity DNA polymerase (Figure 2a), implying an improvement of about 2.1–10.8 times in the cDNA product by the addition of high-fidelity DNA polymerase. Sanger sequencing of the qPCR products confirmed that the high-fidelity DNA polymerase exercised its proofreading activity in a 3′ mismatch on a DNA–RNA duplex. An identical sequence to the RNA template was obtained when the RT reaction contained the high-fidelity DNA polymerase regardless of the use of any one B3-Mut primer (Figure S5b,c), although for only half of the sequences when using aB3-Mut-C primers. These results confirm that the high-fidelity DNA polymerase can exercise its proofreading activity in a 3′ mismatch on a DNA–RNA duplex. It significantly improved cDNA synthesis in the RT reaction, enabling us to design a mismatch-tolerant RT-LAMP assay with excellent tolerance to highly variable target sequences, contrary to conventional LAMP systems.^22,23^

We evaluated the performance of such variant-tolerant assays based on using the HFman probe by testing a series of mutant primers that can form two to three different types of mismatches with the 3′-ends of B3, BIP, and LB, as well as the 5′-end of BIP (Figure 2b–d). The results show that the HFman probe-based RT-LAMP assay generated similar Tt values to those of the wild-type primer, regardless of any one mutant primer (Tt: 9.0–13.6 *vs* 10.6 min) and/or the combination of two or three mutant primers (Tt: 9.2–11.0 *vs* 10.4 min) (Figure 2c,d). These results indicate that the HFman probe-based real-time RT-LAMP assay has high adaptability to highly variable target sequences (e.g., highly variable viral genomic sequences).

### Sensitivity and Specificity of the Multiplex RT-LAMP

**Figure 3:**
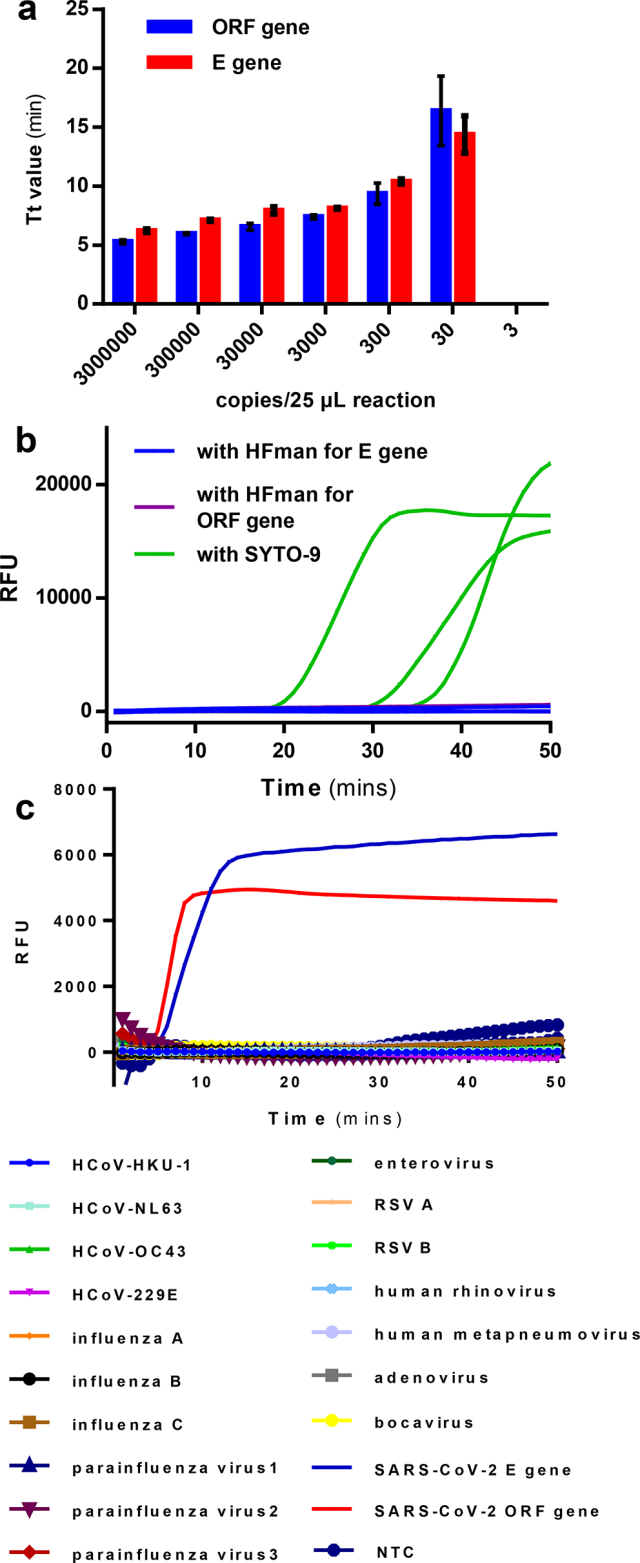
Sensitivity and specificity of the multiplex RT-LAMP assay. (a) Sensitivity of the multiplex RT-LAMP assay for SARS-CoV-2 ORF (blue) and E (red) genes. Time to positive for serially diluted RNA standards of the SARS-CoV-2 ORF and E genes from 3 × 10^6^ copies to 3 copies in 25 μL reactions (average of three technical replicates). Error bars are standard deviations. (b) Specificity experiments of the RT-LAMP assay using RNase-free water (NTC), with SYTO-9 (green) showing non-specific amplification and the HFman probe for ORF (purple) and E genes (blue) showing no amplification. (c) Cross-reactivity of the HFman probe-based multiplex SARS-CoV-2 RT-LAMP assay against 17 common respiratory viruses (HCoV-HKU-1; HCoV-NL63; HCoV-OC43; HCoV-229E; influenza A, B, and C viruses; parainfluenza virus type 1–3; enterovirus; RSV A and B groups; human rhinovirus; human metapneumovirus; adenovirus; and bocavirus). NTC, non-template control. Only the two specific amplifications for ORF (red) and E (blue) genes show a significant increase in the signal.

The sensitivity was determined using a 10-fold serially diluted RNA standard from 10^6^ to 1 copies/μL, each with three replicates, showing the detection of 30 copies of the ORF and E gene RNA within 30 min (Figures 3a and S6). We further measured the limit of detection (LOD) of the multiplex RT-LAMP assay with 10 replicates for decreasing concentrations of RNA (Table 1). The results showed that all 20 reactions (100%) were positive above 120 copies of SARS-CoV-2 RNA. The LOD was estimated at 78 and 115 copies per reaction for the ORF and E genes with a 95% confidence level, respectively (Table 1). As diagnosis of COVID-19 requires a positive test for two different SARS-CoV-2 genes, the overall assay LOD was 115 copies per reaction. Given that the viral load of SARS-CoV-2 in nasopharyngeal swab samples of COVID-19 patients is in the range of 10^3^ to 10^9^ copies per mL,^29−31^ the multiplex RT-LAMP assay was sufficiently sensitive to detect SARS-CoV-2 RNA in clinical situations.^32^ To test whether the HFman-based pipeline improved the specificity of the multiplex RT-LAMP, we performed SYTO-9-based (non-specific DNA binding fluorescent dye) and HFman probe-based multiplex RT-LAMP reactions using RNase-free H_2_O (non-template control, NTC). Non-specific amplification (Tt values: 21.8–38.0) occurred in the RT-LAMP reaction with SYTO-9, but no amplification signal was observed in the reactions with HFman probes (Figure 3b).

**Table 1:** LOD of the Multiplex RT-LAMP for SARS-CoV-2 Detection (See Data Analysis in Methods)

template input (copies/25 μL reaction)	ORF gene (positive/total)	E gene (positive/total)
3000	20/20	20/20
600	20/20	20/20
120	20/20	19/20
24	7/20	10/20
5	3/20	4/20
LOD (copies/25 μL reaction)	78	115

We further validated the specificity of the multiplex RT-LAMP against 17 common respiratory viruses, including HCoV-HKU-1; HCoV-NL63; HCoV-OC43; HCoV-229E; influenza A, B, and C viruses; parainfluenza virus type 1–3; enterovirus; RSV A and B groups; human rhinovirus; human metapneumovirus; adenovirus; and bocavirus, with no amplification signal observed. There was also no amplification of 10 replicates of the NTC within 50 min, confirming the high microbial specificity of the assay (Figure 3c).

To further examine the specificity of the LAMP primers of the ORF and E genes, we performed the sequence analysis of seven human coronaviruses, including SARS-CoV-2, SARS-CoV, MERS-CoV, OC43, HKU1, NL63, and 229E, corresponding to the LAMP primers of the ORF and E genes (Figure S7). Although SARS-CoV-2 shares a relatively high sequence identity when compared to SARS-CoV, the primers of ORF and E correspond to gaps or insertions in the genome of MERS-CoV and the other four common human coronaviruses OC43, 229E, NL63, and HKU1. The results indicate that the primers of ORF and E are highly specific to SARS-CoV-2.

### Establishment of the RNA Extraction-Free Multiplex RT-LAMP Assay for SARS-CoV-2

In conventional testing workflows, viral RNA is extracted from clinical samples, which prevents the implementation of testing at the point of care and leads to delays in results. An RNA extraction-free detection assay not only facilitates the diagnosis of SARS-CoV-2 infection but also avoids potential risks of exposure for healthcare staff during sample preparation.^14^ Because of the infectious nature of SARS-CoV-2, it was not possible to use clinical samples directly for development, so instead we spiked the prepared RNA standard into RNase free water (see Supporting note results and Figure S8a for buffer optimization) in throat swab samples collected from healthy individuals to simulate the real samples using a heat inactivation step (95 °C for 10 min) before the RT-LAMP reaction (Figure S9a).

The amplification was monitored using the real-time PCR machine or observed by the naked eye (after 50 min) under blue illumination (Figure S9b). To verify the feasibility of the RNA extraction-free assay, we first used the treated simulated samples that contained 200 copies each of the ORF and E gene RNA to perform the triplex RT-LAMP. All three targets could be detected in the single-tube reaction (Figure S8b). To optimize the volume of the template input and determine the detection sensitivity of the RNA extraction-free multiplex RT-LAMP assay, 25 μL multiplex RT-LAMP reactions were performed with 2, 4, 6, 8, 10, and 12 μL inputs of 50 copies/μL and 5 copies/μL simulated samples. The results showed that the SARS-CoV-2 RNA could be detected by the RNA extraction-free multiplex RT-LAMP assay with direct sample inputs of 2–12 μL when the viral load was over 5 × 10^4^ copies RNA per mL (Figure S8c). When the viral load was over 5 × 10^3^ copies per mL, SARS-CoV-2 could be detected with a sample of 6–12 μL (equivalent to 30–60 copies per 25 μL reaction) (Figure S8d).

### Evaluation of the Multiplex RT-LAMP Using Extracted Clinical Samples

To verify the diagnostic accuracy of the multiplex RT-LAMP assay, 190 nasopharyngeal swab (NP) samples were extracted and analyzed (Figure S10). For comparison, two approved commercial RT-qPCR kits were used for the first batch of 99 clinical samples (Figure S11), and only the BioPerf kit was used for the second batch of 91 samples. Among 190 NP samples, the BioPerf kit detected 87 samples positive for both ORF and N genes of SARS-CoV-2, and an additional three samples positive only for the N gene with high Ct values (35.8–39.2) (Figure S12). The multiplex RT-LAMP assay detected 81 samples positive for both the ORF and E genes of SARS-CoV-2 and an additional five samples positive only for E or ORF (Figure S12). Among 100 double-negative samples by the BioPerf RT-qPCR assay, one sample (no. 31) was detected as ORF gene positive by the multiplex RT-LAMP assay and further confirmed as ORF gene positive by another RT-qPCR assay (BioGerm kit) (Figure S11), thereby being considered as a weak positive sample. Using a single gene as an output (samples positive for either ORF or N genes by the RT-qPCR assay), 91 samples (including sample 31) were identified as SARS-CoV-2 positive. The BioPerf RT-qPCR assay and the multiplex RT-LAMP assay detected 90 and 86 positive samples, showing the sensitivities of 98.9 and 94.5%, respectively (Table 2). 22 SARS-CoV-2 variants/lineages were identified from the 53 positive samples, with the alpha variant and the B.1 lineage as the most commonly identified variants/lineages (Table S2). The RT-LAMP assay detected all these variants/lineages except one B.1 lineage. All the other 99 samples were detected as SARS-CoV-2 negative for both genes (ORF and N or E genes) of SARS-CoV-2 by the BioPerf RT-qPCR assay and the multiplex RT-LAMP assay, showing 100% specificity. The consistency between the BioPerf RT-qPCR assay and the multiplex RT-LAMP assay was 96.8% (184/190) (Table 2).

**Table 2:** Comparison of the Multiplex RT-LAMP Assay with a Commercial RT-qPCR Assay

method	the BioPerf RT-qPCR assay						
		a single gene as output			ORF gene		
the multiplex RT-LAMP		positive	negative	total	positive	negative	total
	positive	85	1^a^	86	81	1^a^	82
	negative	5	99	104	6	99	105
	total	90	100	190	87	100	187
sensitivity		94.5%			93.2%		
specificity		100%			100%		
consistency		96.8%			96.3%		

a This sample was confirmed as ORF gene-positive by the multiplex RT-LAMP and the BioGerm RT-qPCR assay.

There were five false-negative samples by the multiplex RT-LAMP assay. Of them, three (samples 60, 69, and 76) were single gene-positive (N gene), and two (samples 22 and 58) were double gene-positive by the RT-qPCR assay (Figure S12). Four of the samples had very high Ct values (33.8–39.2 for the N gene and 37.1 for the ORF gene) in the RT-qPCR assay, implying a very low viral load. Furthermore, there were five single gene-positive samples by the multiplex RT-LAMP assay. Three samples (19, 53, and 59) had Ct values of 34.5–38.3 for the N gene and 33.2–35.2 for the ORF gene, and the other two (58 and 74) had relatively low Ct values (26.5–27.8 for the N gene and 24.1–25.2 for the ORF gene).

**Figure 4:**
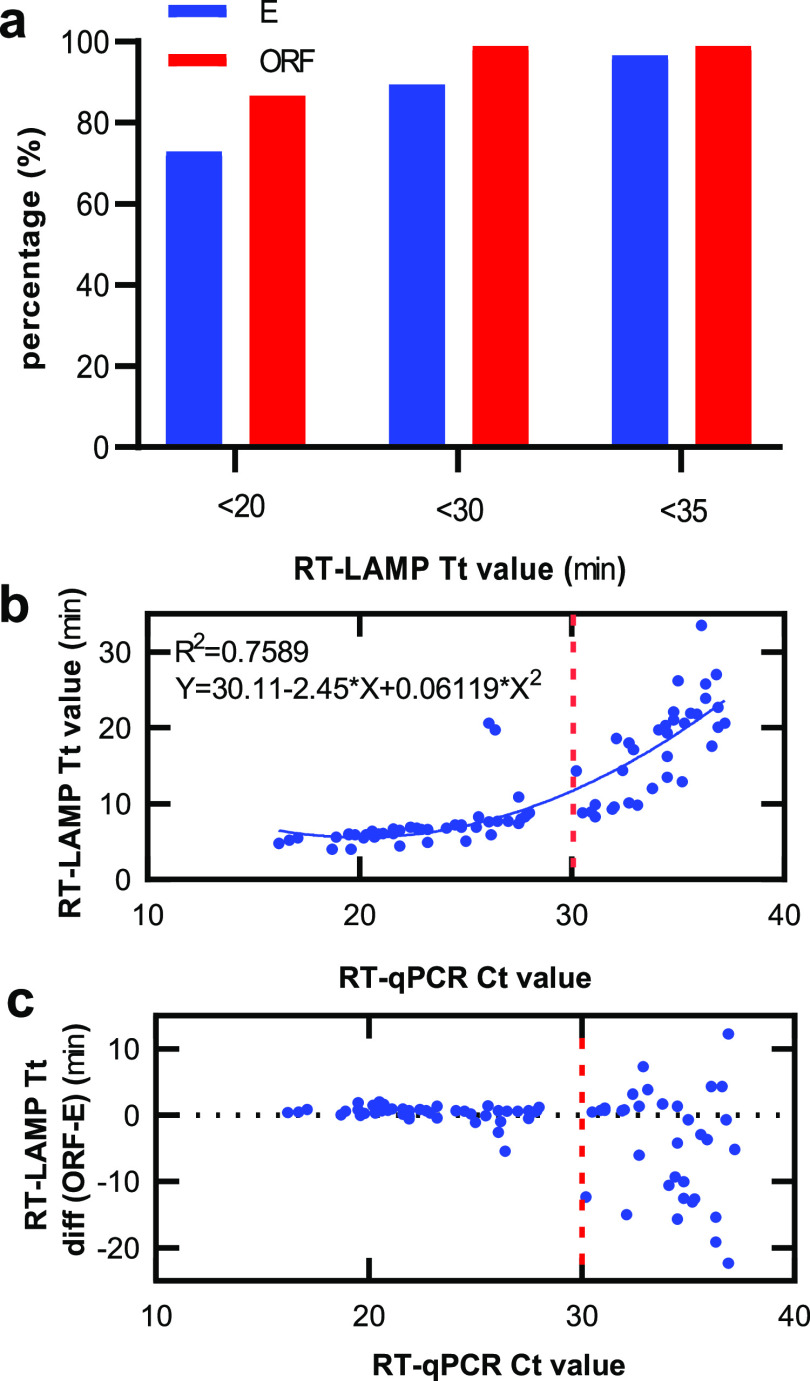
Clinical validation of the multiplex SARS-CoV-2 RT-LAMP using extracted RNA from nasopharyngeal swab samples. (a) Percentage (%) of Tt values of the multiplex RT-LAMP assay less than 20, 30, and 35 min. (b) Scatter plot of the ORF gene Tt values of the multiplex RT-LAMP and the ORF gene Ct values of the commercial RT-qPCR assay on 81 NP samples. (c) Scatter plot of the Tt difference (Tt-Diff) between the ORF and E genes by the multiplex RT-LAMP and the Ct values of the ORF gene by the commercial RT-qPCR assay on 81 NP samples. * only one of 99 negative results (samples 47, 61, 75, and 95–190) is shown.

The multiplex RT-LAMP assay was fast as most reactions were completed within 35 min (100% for ORF and 96.5% for E) (Figure 4a). In particular, about 86.6 and 98.8% of reactions had Tt values less than 20 and 30 min for ORF and about 72.9 and 89.4% for E genes, respectively. Because both the RT-qPCR and the RT-LAMP assays target the ORF gene, we further analyzed the relationship of the RT-qPCR Ct value and the multiplex RT-LAMP Tt values for the ORF gene. The Tt value remained relatively stable when the Ct values were less than 30 and increased sharply along with the increase in Ct values when the Ct values were more than 30, respectively (Figure 4b).

Furthermore, the multiplex RT-LAMP generated very consistent Tt values for both ORF and E genes when the Ct values were less than 30 but more variable Tt values between ORF and E genes when the Ct values were more than 30 (Figure 4c), indicating that a low viral load might cause large variation of Tt values between genes in multiple RT-LAMP assays.

### Clinical Evaluation of the Multiplex RT-LAMP without Extraction

As stated, the use of complex sample preparation processes for the extraction of nucleic acids from clinical samples prevents the use of highly sensitive molecular techniques at the point of care. We further evaluated the performance of our multiplex RT-LAMP assay when using clinical samples directly, that is without extraction. We used 49 SARS-CoV-2 positive NP samples identified by the BioPerf RT-qPCR assay. Two approved commercial viral transport media (VTM) (VTM-KJ: 156-102B, Kangjian Medical, Jiangsu, China; VTM-CR: CR24180 Cienry, Zhejiang, China) were previously used to collect the NP samples. Because of the inhibition of VTM on the reaction (Figure S8a and Supporting note results), only 6 μL of NP-VTM samples (about equal to 2.4 μL extracted RNA, half of the RNA amount used in the RT-qPCR assay) was directly inputted in each 25 μL multiplex RT-LAMP reaction. Using a single gene as the output, 33 samples were detected as positive by the extraction-free multiplex RT-LAMP pipeline (Figure S13a), showing a sensitivity of 67.3%. A sample is considered positive for SARS-CoV-2 by RT-qPCR when the Ct < 40 (and there is a clear amplification curve). When Ct values are under 35, RT-qPCR provides a useful correlation with viral load (e.g. ∼10^4^ copies/mL and 10^3^ copies/mL for Ct 30 and 35, respectively). When the Ct values of the samples were less than 30, the sensitivity increased to 90.6% (Figure S13b). The NP-VTM-CR yielded sensitivities of 86.4% overall and 94.1 and 95.0% for samples with RT-qPCR Ct values of less than 30 and 35, respectively, substantially higher than those of NP-VTM-KJ (51.9, 86.7, and 63.6%), Figure S13b. These results suggest that VTM-CR is more suitable to the direct multiplex RT-LAMP assay than VTM-KJ and can be widely used for rapid, sensitive, specific point-of-care diagnosis and/or mass screening of SARS-CoV-2, and other emerging and re-emerging respiratory viruses (e.g., influenza virus). However, it should be noted that because of the relatively smaller amount of the template input, the direct/extraction-free multiplex RT-LAMP yielded substantially higher Tt values for both ORF and E genes than with the extracted RNA (Figure S14).

## Discussion

Highly transmissible human coronavirus, SARS-CoV-2, has caused a global pandemic with a significant economic burden, including at least 272 million infections and 5.3 million deaths.^33−35^ Like symptomatic COVID-19 patients, both pre-symptomatic and asymptomatic individuals are infectious,^36,37^ with the majority of new infections being caused by “silent transmission”.^1,2^ Early diagnosis and/or screening to identify pre-symptomatic and asymptomatic individuals is critical to the future containment of the pandemic and requires a simple, rapid, sensitive, and accurate diagnostic assay. Although the RT-qPCR methodology is accepted as the golden standard for the diagnosis of SARS-CoV-2 infection, it needs to be performed in well-found laboratories with specialized equipment and trained healthcare staff. As previously stated, positive results for the two different genes of SARS-CoV-2 are required for the diagnosis of COVID-19. To facilitate the diagnosis and screening of SARS-CoV-2 infection, we have developed a simple, rapid, variant-tolerant, and sensitive point-of-care assay that does not require specialized equipment or sample preparation.^24,25,38,39^

One major reason why LAMP is considered inferior to qPCR is its specificity, accuracy, and multiplexing capacity, which are often reduced through non-specific amplification and/or by non-specific interferents (e.g., non-specific double-stranded DNA binding dyes) (Table S3).^6,15,40,41^ Furthermore, the low detection accuracy of LAMP is also associated with its high vulnerability to mismatches between a large number of LAMP primers and the targets.^22,23^ In this study, we developed an HFman probe-based real-time LAMP method to overcome many of these shortcomings of LAMP, using high-fidelity DNA polymerase to recognize and cleave the HFman probe and release a fluorescence dye from its quenched counterpart. Because the HFman probe can be labeled by different fluorophores and because the specific binding of the HFman probe to the target sequence is a prerequisite for the recognition and cleavage by high-fidelity DNA polymerase to release fluorescent signal, our LAMP system showed excellent specificity and enabled the multiplex detection of different targets in a single-tube reaction.

We show that high-fidelity DNA polymerase efficiently removes mismatched bases between LAMP primers and templates, greatly improving the amplification efficiency of the LAMP method and its performance in the detection of highly variable viruses.^22−24,38^ We also demonstrate that the high-fidelity DNA polymerase can recognize and remove the 3′ mismatched base on a DNA–RNA duplex, with the inclusion of the enzyme in the RT reaction increasing cDNA products, giving a consistent amplification efficiency (similar Tt values) for a number of variants carrying one or more mutations to the 3′-end of the primer sequences. Table S2 provides information (where available) on the variants present in the clinical samples analyzed in this work, showing the capability of our assay to detect all of them, including alpha (B.1.1.7) and delta (B.1.617.2). Although gamma (P.1) and omicron (B.1.1.529) variants form one mismatch at the middle of the ORF gene LF primer and the 3′-end third last site of the E gene F2 primer, respectively (Figure S15), the variant-tolerance feature of the system enables effective detection of these variants.^22^ These results indicate that our HFman probe-based LAMP method is especially suitable for the detection of highly variable RNA viruses that contribute to most emerging and re-emerging infectious diseases (e.g., AIDS, ZIKA, influenza, and COVID-19) and which have shown “diagnostic escape”.^3,42^ Our real-time RT-LAMP achieves higher sensitivities and specificities and better tolerance to highly variable sequences, as well as the capacity for single-pot multiplex detection (Table S3) when compared to the mismatch-tolerant and conventional LAMP methods.

Compared to the gold standard qPCR method, our real-time LAMP method has comparable sensitivity and specificity, with the capacity for multiplex detection and fast amplification (<30 min) (conferring advances over the existing RT-qPCR methods) (Table S3). The cost of our method is also low (total cost of $0.5–1 in the singleplex–multiplex format) when compared to other formats.

As the HFman probe shares the same sequence as the LF or LB primer, there is no need to re-design additional probes, implying that any current LAMP assay can be easily updated into the high-specific HFman probe-based real-time pipeline and is ready to be developed into a single-tube multiplex detection by combining primers and probes for different targets. As the majority of the amplification curves reach a plateau after 20–30 min, a 30 min reaction time is recommended for detection of clinical samples, or the appearance of a clear amplification curve within 30 min is used as a determination of positive for SARS-CoV-2 or other pathogens.

## Conclusions

Using the HFman probe-based multiplex system, we developed a rapid, sensitive, and specific RT-LAMP assay for the simultaneous detection of two different genes (ORF and E) of SARS-CoV-2. Our SARS-CoV-2 assay shows high sensitivity (94.5%), specificity (100%), and consistency (96.8%) against a commercial RT-qPCR assay on purified RNA. It also has a high adaptability to variable target sequences. Importantly, we demonstrated that the multiplex RT-LAMP assay can be delivered in an extraction-free format, which can be completed within 45 min using a simple heat block (Figure S9) or other low-resource heating methods.^43^

## Materials and Methods

### Preparation of the RNA Standard

The PUC-57 plasmids containing the ORF 1ab gene and E gene of SARS-CoV-2 were synthesized by Sangon Biotech (Shanghai, China). To prepare the RNA standard, ORF 1ab and E genes were first amplified using specific PCR primers (Table S1) containing a T7 promoter with PUC-57 plasmids as a template. The PCR amplicons were extracted and purified with a commercial DNA extraction kit (Monarch, NEB). RNA was synthesized *in vivo* using HiScribe T7 Quick High Yield RNA Synthesis Kit (New England Biolabs, England) and treated by DNase I to remove the DNA template. The obtained RNA was extracted and purified using alcohol and LiCl. The concentration of the RNA standard was quantitated using Qubit 4.0 (Thermo Fisher Scientific).^24^

Detailed methods for reverse transcription, qPCR, and Sanger sequencing are provided in the Supporting Information.

### Multiplex RT-LAMP Reaction

The LAMP reaction was conducted in 25 μL, containing 1 × isothermal amplification buffer, 8 mM MgSO_4_, 1.8 mM dNTP, 8 U Bst 4.0 DNA/RNA polymerase (Haigene, China), 0.15 U high-fidelity DNA polymerase, 0.1 μM each of F3 and B3, 1.0 μM each of FIP and BIP, 0.4 μM LF (or 0.2 μM LF and 0.2 μM HFman probe), and 0.4 μM LB (or 0.2 μM LB and 0.2 μM HFman probe). After optimization (Figure S4) for the multiplex assay, the primer concentrations of the ORF and E genes were halved, while the primer concentrations of the β-actin gene were kept unchanged. The primers of ORF and E genes were described in previous studies.^25,44,45^ The primers of the β-actin gene were designed using PrimerExplorer V5. All the primer information is listed in Table S1. The reaction was performed at 64 °C for 50 min in a CFX 1000 touch real-time PCR detection system (Bio-Rad Laboratories, USA) for real-time monitoring by collecting the fluorescence signal every minute for three channels (CY5, FAM, and HEX channels). The endpoint visual image was photographed using a smartphone or a portable device with 465 nm light.

### Sensitivity and Specificity of the Multiplex RT-LAMP

10-fold serial dilutions of the RNA standard from 10^6^ copies/μL to 1 copy /μL were used to determine the sensitivity, and each assay was repeated in triplicate. The specificity was evaluated using 17 common respiratory viruses, including influenza A, B, and C viruses; parainfluenza viruses type 1–3, enterovirus; RSV A and B groups; HCoV-HKU-1; HCoV-NL63; HCoV-OC43; HCoV-229E; human rhinovirus; human metapneumovirus; adenovirus; and bocavirus. Nucleic acids were extracted from positive throat swab samples of children with acute respiratory tract infections or from the virus stock stored in our laboratory.

### Development of an RNA Extraction-Free Multiplex RT-LAMP Assay for SARS-CoV-2

To simulate clinical samples for our development phases, we artificially prepared samples by spiking RNA standards to throat swab samples collected from healthy volunteers. Swabs were placed into 1.5 mL of buffer that contained nuclease-free water and 1 U/μL of RNase inhibitor (RNasin Plus, Promega) and were vortexed. 200 μL aliquots were spiked to achieve final concentrations of 5 and 50 copies/μL of RNA standards. The samples were then inactivated at 95 °C for 10 min.^46,47^ Multiplex real-time LAMP was performed using 2, 4, 6, 8, 10, or 12 μL of the sample input in a 25 μL reaction.

### Clinical Evaluation of the Multiplex SARS-CoV-2 RT-LAMP Assay

A total of 190 nasopharyngeal swab (NP) samples were collected from individuals entering China from overseas from September 2020 to June 2021. The swabs were transferred to a 3 mL viral transport medium (156-102B, Kangjian Medical, Jiangsu, China; or CR24180 Cienry, Zhejiang, China). Viral RNA was extracted from 200 μL NP samples using an RNA extraction kit (SDK60104, BioPerfectus Technologies, Taizhou, China) and eluted in 80 μL of nuclease-free water for immediate use or for storage at −80 °C. To evaluate the performance of the multiplex SARS-CoV-2 RT-LAMP assay, two commercial RT-qPCR kits (BioPerfectus Technologies, Taizhou and BioGerm, Shanghai, China) that target ORF and N genes of SARS-CoV-2 were used. Both RT-qPCR kits were approved by the National Medical Products Administration of China. To perform the evaluation, the same amount of extracted RNA (5 μL) was added to each 25 μL reaction of the multiplex SARS-CoV-2 RT-LAMP assay and the two RT-qPCR assays. The reactions were performed according to the manufacturer’s instructions using Light Cycler 480 (Roche, Switzerland) or Applied Biosystems ABI 7500 (Thermo Fisher Scientific, USA). 49 positive NP samples (BioPerf RT-qPCR assay) were also used and directly stored in different transport media (22 samples with VTM-KJ: 156-102B, Kangjian Medical, Jiangsu, China; and 27 samples with VTM-CR: CR24180 Cienry, Zhejiang, China). 6 μL was added to 25 μL reactions.

### Data Analysis

To determine the LOD of the assay, 25 μL reactions with fivefold serial dilutions of the RNA standard from 3000, 600, 120, and 24, to 4.8 copies were performed. Each dilution was tested in a set of 10 replicates. The LOD was defined as a 95% probability of obtaining a positive result using probit regression analysis with SPSS 17.0 software.^48^ The bar graphs and scatter plot were drawn using GraphPad Prism 6.

### Ethics Statement

The study was approved by the Nantong Third Hospital Ethics Committee (E2020002). Written informed consent was obtained from all participants.

## Supporting Information Available

Influence of the viral transport medium (VTM) on the real-time RT-LAMP amplification; optimization of the concentrations of the HFman probe and its corresponding loop primer with the same sequence; optimization of the amount of the primer and probe in the multiplex RT-LAMP for SARS-CoV-2. All the raw data associated with this article are available open access in the University of Glasgow repository at http://dx.doi.org/10.5525/gla.researchdata.1185 (PDF)
