# Supplementary material for: Multiplex, Real-Time, Point-of-care RT-LAMP for SARS-CoV-2 Detection Using the HFman Probe
Source: ACS Sens. 2022 Feb 22;7(3):730–9. doi: 10.1021/acssensors.1c02079 (PMC8887655; doi:10.1021/acssensors.1c02079)
Supplement: Supplementary file 1 — se1c02079_si_001.pdf [file se1c02079_si_001.pdf]

Supporting Information

Multiplex, variant-tolerant, real-time, point-of-care RT-LAMP for SARS-CoV-2 detection using HFman probe

Yajuan Dong [1, 2, #], Yongjuan Zhao[2, #], Shenwei Li[3], Zhenzhou Wan[4], Renfei Lu[5], Xianguang Yang[1], Guoying Yu[1], Julien Reboud[6], Jonathan M. Cooper[6]*, Zhengan Tian[3]*, Chiyu Zhang[2]*

Affiliations:
[1] College of Life Sciences, Henan Normal University, Xinxiang 453007, China
[2] Shanghai Public Health Clinical Center, Fudan University, Shanghai 201508, China
[3] Shanghai International Travel Healthcare Center, Shanghai 200335, China
[4] Medical Laboratory of Taizhou Fourth People's Hospital, Taizhou 225300, China
[5] Clinical Laboratory, Nantong Third Hospital Affiliated to Nantong University, Nantong 226006, China
[6] Division of Biomedical Engineering, University of Glasgow, G12 8LT Glasgow, United Kingdom

# These authors contributed equally to this study.

*Corresponding authors:

Chiyu Zhang, PhD, Shanghai Public Health Clinical Center, Fudan University, 2901 Caolang Road, Shanghai 201508, China. E-mail address: zhangcy1999@hotmail.com

Or Zhengan Tian, Shanghai International Travel Healthcare Center, Shanghai 200335, China. E-mail address: tianzhenganciq@163.com

Or Jonathan M. Cooper, Division of Biomedical Engineering, University of Glasgow, G12 8LT Glasgow, United Kingdom. E-mail address: Jon.Cooper@glasgow.ac.uk

Supporting note Method

Supporting note Results

Supporting Figures S1-15

Supporting Tables S1-S3

Supporting note - Method.

Reverse transcription, qPCR and Sanger sequencing.

Reverse transcription (RT) was carried out using the WarmStart RT Reverse Transcriptase (New England Biolabs, American) with adaptor-B3 (aB3) primer in T100™ Thermal Cycler (Bio-Rad, Germany) instrument at 64 °C for 50 min. The 20 µL of RT reaction contains 1x isothermal amplification buffer, 6 mM $MgSO_4$, 1.8 mM dNTP, 7.5 units of WarmStart RT (New England Biolabs, American), 0.5 µM aB3 primer, with and without 0.15 U High-fidelity DNA polymerase (New England Biolabs, American), and 3 µL RNA standard (10^5 copies/µL). Obtained cDNA was subjected to qPCR assay that was performed using the FastFire qPCR PreMix Kit (Tiangen Biotech, China) with F3 and adaptor primers. The 20 µL of qPCR reaction mixture containing 1× Supermix buffer, 300 nM each of primers, and 1.0 µL cDNA was run in a CFX 1000 Touch Real-Time PCR Detection System (Bio-Rad Laboratories, USA) with cycling condition of 1 min at 95 °C for initial denaturation, 40 cycles of 5 s at 95 °C for denaturation and 15 s at 60 °C for annealing and extension. The qPCR products were separated by electrophoresis on a 1% agarose gel, and purified with Monarch DNA Gel Extraction Kit (New England Biolabs, USA). Sanger sequencing of purified qPCR products was by Sangon Biotech (Shanghai, China).

Supporting note - Results.

The influence of viral transport medium (VTM) on the real-time RT-LAMP amplification

To develop an RNA extraction-free/direct RT-LAMP assay directly using nasopharyngeal swabs (NP) (Fig. S9), we firstly measured the influence of viral transport medium (VTM) and RNase-free water on the real-time RT-LAMP amplification. The NP samples collected from healthy individuals were put into VTM (NP-VTM) and RNase-free water (NP-water), and then heat-inactivated at 95 °C for 10 min before use [1]. The results show that the NP-VTM sample had a dose-dependent inhibition on the real-time RT-LAMP amplification, especially when the sample input was more than 6 µL in 25 µL reaction; whilst the NP-water had less or no influence on RT-LAMP amplification even when the sample input reached up to 12 µL (48% of total reaction volume) in 25 µL reaction (Supplementary Fig. S8). These indicate that RNase-free water might be optimal buffer for the collection of NP samples from COVID-19 patients and other respiratory tract infected individuals in future.

Supporting Figures

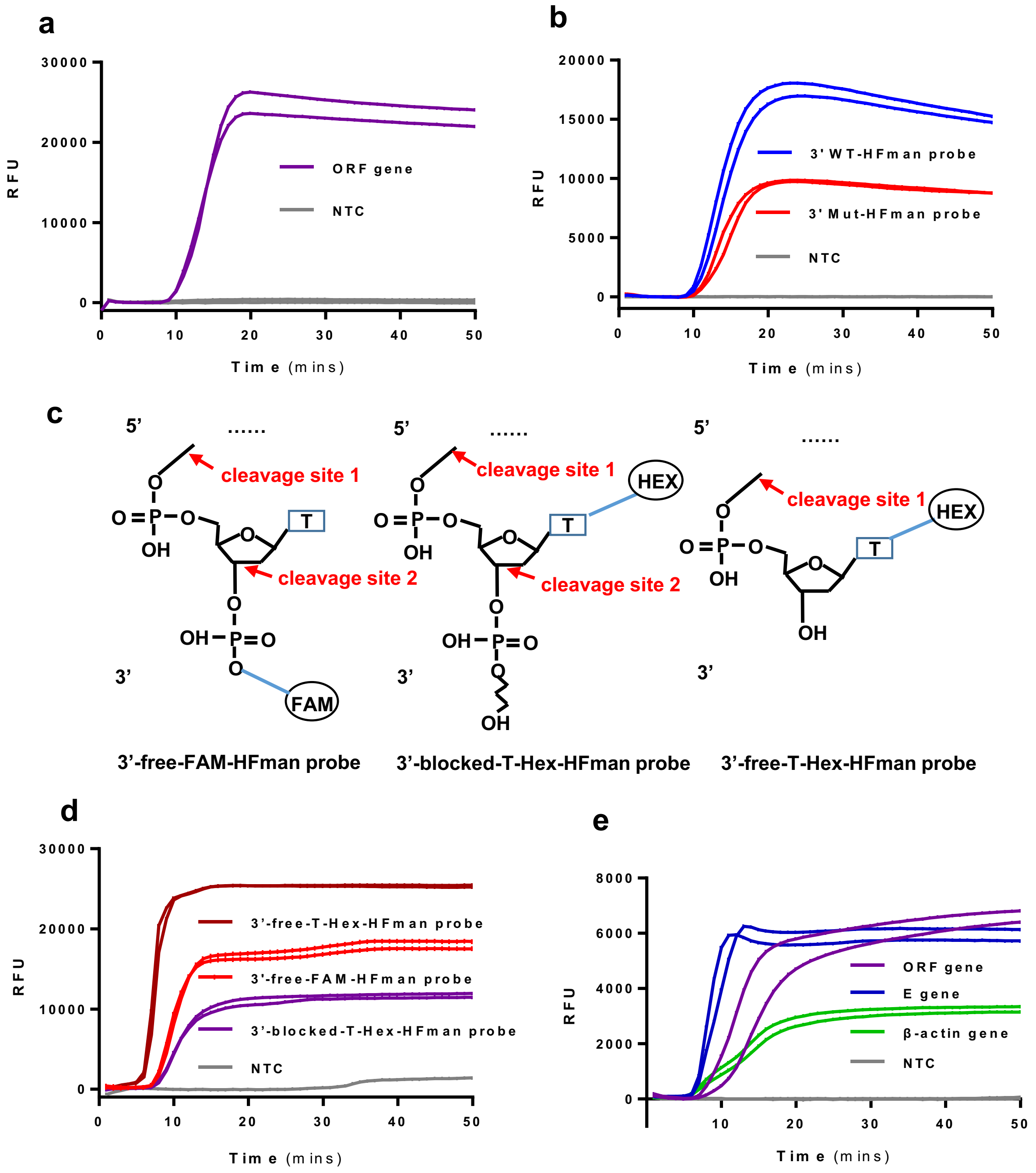

Fig. S1. Establishment of the multiplex real-time RT-LAMP assay. (a) Detection of the SARS-CoV-2 ORF gene using a specific HFman probe. **(b)** Performance comparison of the real-time RT-LAMP between the wild-type HFman (HFman-WT) probe (3’end base A) and the mutant HFman (HFman-Mut) probe (3’end base G) for ORF gene. **(c)** The structures of three HFman probes for SARS-CoV-2 E gene (3’-free-FAM-HFman probe, 3’-blocked-T-Hex-HFman probe and 3’-free-T-Hex-HFman probe) and their potential cleavage sites by high-fidelity DNA polymerase (arrows). All three probes share the identical sequence with a base “T” at the 3’-end. The 3’-free probes have a free 3’-OH. The 3’-OH of the 3’-blocked-T-Hex-HFman probe was blocked by a C3 spacer. **(d)** Real-time RT-LAMP with the three HFman probes, described in (c), for SARS-CoV-2 E gene (namely, 3’-free-FAM-HFman probe, 3’-blocked-T-Hex-HFman probe and 3’-free-T-Hex-HFman probe). **(e)** Simultaneous detection of ORF and E genes of SARS-CoV-2, and human house-keeping gene β-actin as a multiplex RT-LAMP. All RT-LAMP reaction were performed twice (two curves of the same condition) with 3×10^5 copies template input of ORF gene RNA (panels a and b), E gene RNA (panel d) and ORF, E and β-actin gene RNA (panel e). NTC: non-template control (RNase-free water).

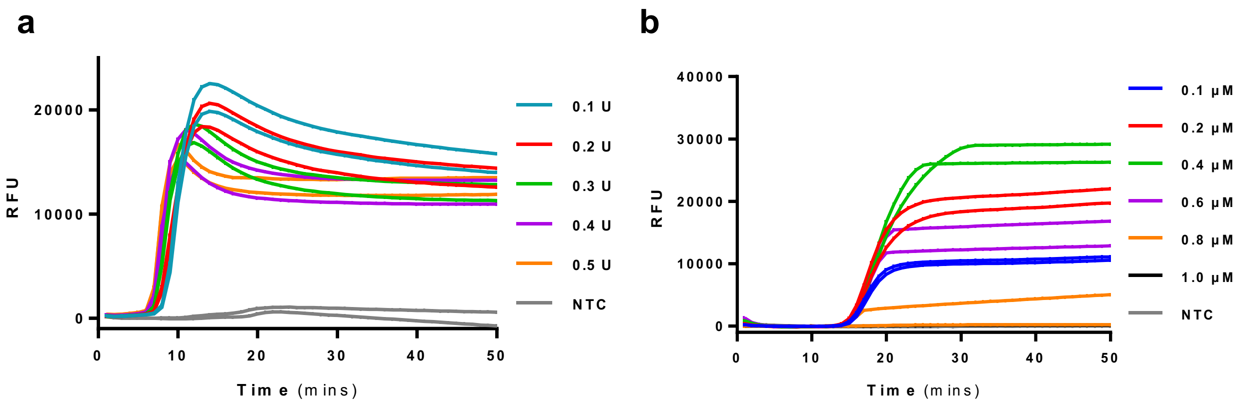

Fig. S2. Optimization of the amount of High-fidelity DNA polymerase (a) and the concentration HFman probe (b). 3×10^{5}copies of ORF and E gene RNA standards were used for the 25μL RT-LAMP reaction.

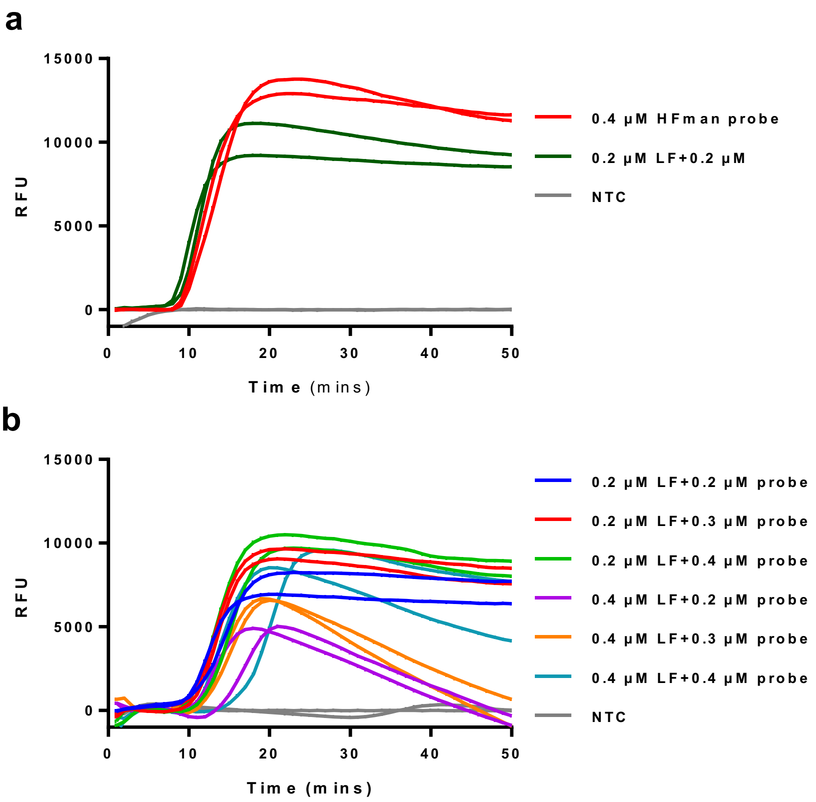

Fig. S3. Optimization of the concentrations of the HFman probe and its corresponding loop primer with the same sequence. (a) Performance comparison of the real-time RT-LAMP with 0.4 μM HFman probe only and with 0.2 μM HFman probe and 0.2 μM LF primer. (**b)** Optimization of the ratio of HFman probe and its corresponding LF primer. 3×10^{3}copies of ORF and E gene RNA standards were used for the 25 μL RT-LAMP reaction.

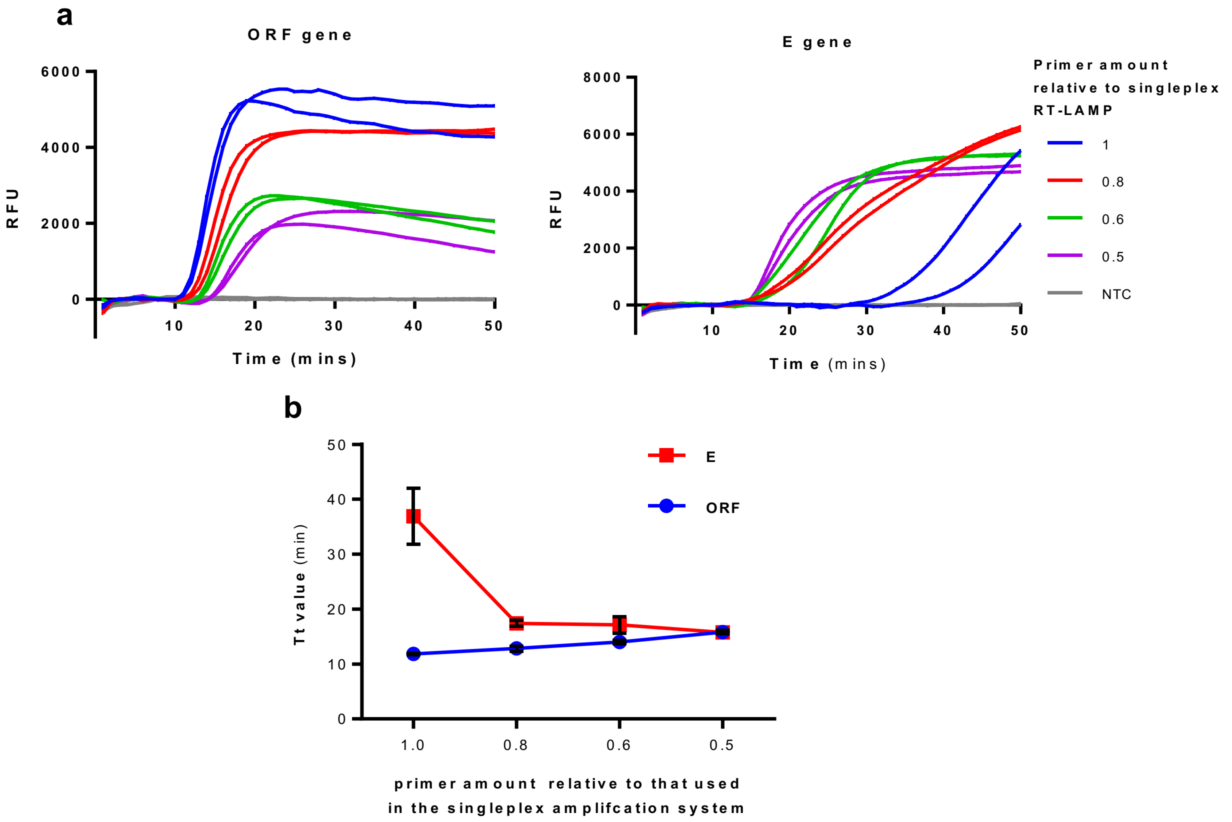

Fig. S4. Optimization of the amount of the primer and probe in the multiplex RT-LAMP for SARS-CoV-2. (a) Optimization of the amount of the primer/probe set. (**b)** Comparison of the Tt values of the ORF and the E gene in different amount of the primer/probe set. 3×10^3copies of ORF and E gene RNA standards were used for the 25μL RT-LAMP reaction. The mean of Tt values are shown with standard deviation (SD). NTC: non-template control.

a

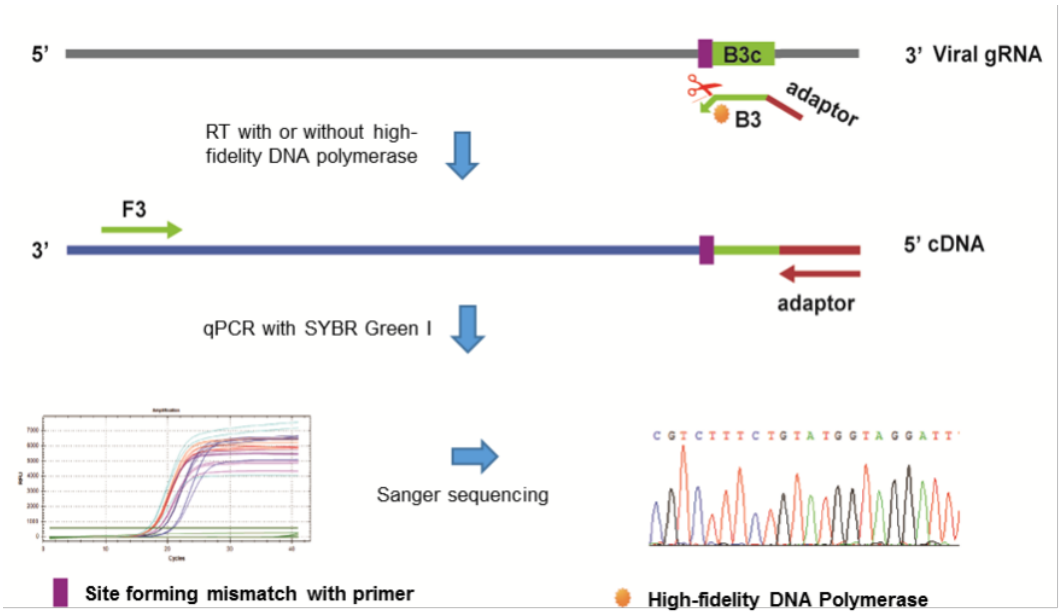

b

RT reaction	Sanger sequencing
aB3-WT	CGTCTTTCTGTATGGTAGGATT
aB3-Mut-A	.....................A
aB3-Mut-A with Q5	....
aB3-Mut-C	C
aB3-Mut-C with Q5	.....................C/T
aB3-Mut-G	.....................G
aB3-Mut-G with Q5	....

c

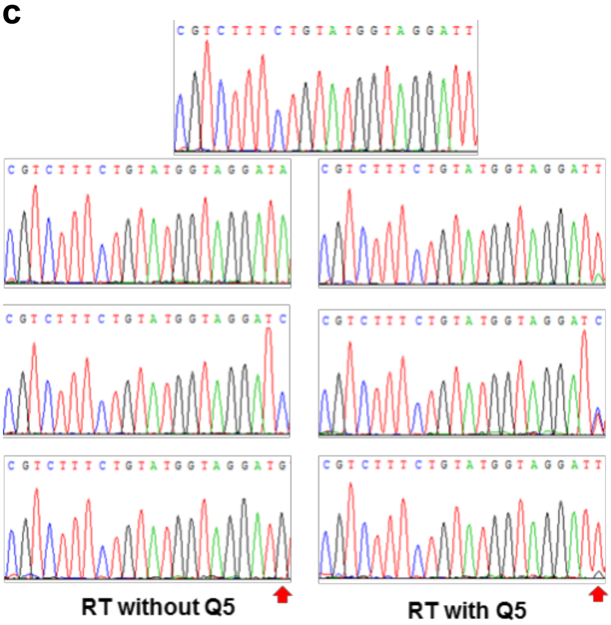

Fig. S5. Functional verification of high-fidelity DNA polymerase to cleave 3' mismatches in reverse transcription (RT) reaction. (a) Strategy for functional verification of mis-match tolerant RT-LAMP. Sequences (b) and chromatograms (c) of different qPCR products by Sanger sequencing to verify high-fidelity DNA polymerase to cleave 3' mismatches in reverse transcription (RT) reaction.

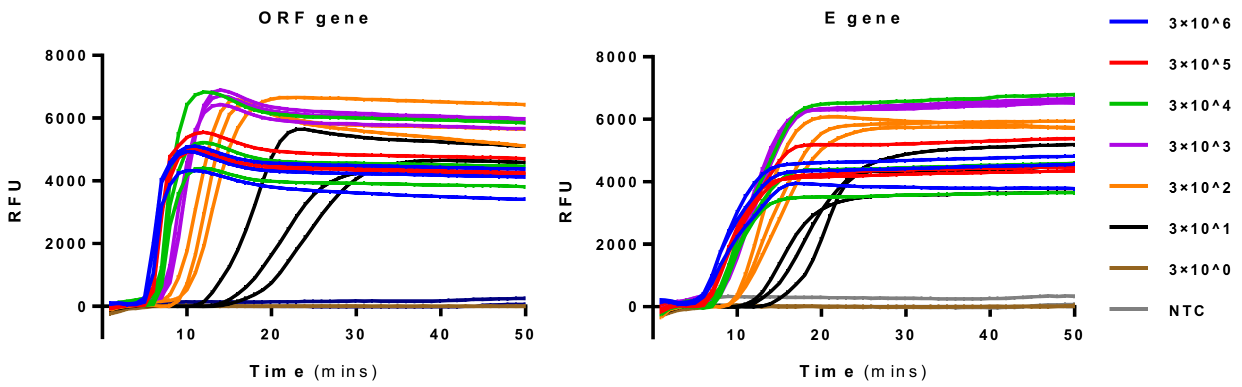

Fig. S6. Amplification curves of multiplex RT-LAMP assay for serially diluted SARS-CoV-2 ORF and E gene serially diluted RNA from 3×10^6 copies to 3 copies.

```
ORF gene
Primer location(5' -3' ) F3                                                                   F2
SARS-CoV-2          TGCAACTAATAA--------------------------------------------------AGCCACG    AAATACCTGGTGTATACGTTGTC
SARS-CoV            G..T..A.CC..--------------------------------------------------GA.A...    ...C..T......T.........
MERS-CoV            A...T..T..G.TACTAATCTTAATAAGTTCAATAGAGCTAGTTTGCGTCAAATTTTTGACGT....C.C    CG.A.ATAAA.TC.C..C..TGA
HCoV-229E           ---------------------------------------------------------------------    -----------------------
HCoV-OC43           ---------------------------------------------------------------------    -----------------------
HCoV-NL63           ---------------------------------------------------------------------    -----------------------
HCoV-HKU1           ---------------------------------------------------------------------    -----------------------
                 B1
SARS-CoV-2       GAC---------------------------------------------------------------------------GCGCAGGGAATGGATAA
SARS-CoV         ...---------------------------------------------------------------------------A.A..A.......C..
MERS-CoV         ..AATGACAATTGTCAAATGTAAGGGTTTAAATAAACCTTTCGTGAAGGACAATGTCAGTTTCGTTGCTGATGACTCAGGTA.T.CC.TTG.T..GT.
HCoV-229E        -------------------------------------------------------------------------------------------------
HCoV-OC43        -------------------------------------------------------------------------------------------------
HCoV-NL63        -------------------------------------------------------------------------------------------------
HCoV-HKU1        -------------------------------------------------------------------------------------------------
                F1                          LF                         LB                        B2                       B3
SARS-CoV-2      ATTCGTTTGATGTACTGAAGTCAGA   TGGAGCACAAAACCAGTTGAAACA   TCTTGCCTGCGAAGATCTAAAAC   AGTCTCTGAAGAAGTAGTGGAA   AATCCTACCATACAGAAAGACG
SARS-CoV        ....A.....A..T...GCAGT...   .....T.....G.....A..T..T   ......T..T...AG..A.C...   CAC...................   .................G..A.
MERS-CoV        C.GTAGA..TG...GCACTTCA.C.   GT.GAGT.T.C..........C.T   ...GT.TAAA.........C.TA   .T.G.A..T...CCCTAA.T.T   C.AGTC.TTG.CTTA......A
HCoV-229E       -------------------------   ------------------------   -----------------------   ----------------------   ----------------------
HCoV-OC43       -------------------------   ------------------------   -----------------------   ----------------------   ----------------------
HCoV-NL63       -------------------------   ------------------------   -----------------------   ----------------------   ----------------------
HCoV-HKU1       -------------------------   ------------------------   -----------------------   ----------------------   ----------------------

E gene
                         F3                     F2                       F1                           B1
Primer location(5' -3' ) AGCTGATGAGTACGAACTT    ATTCGTTTCGGAAGAGACAG     ACTTCTTTTTCTTGCTTTCGTGGT     TTGCTAGTTACACTAGCCATCCTTA
SARS-CoV-2               ..AAAG.............    ...............A....     ........................     ........C................
SARS-CoV                 TTGGACAT.TGGAA...GA    .CC.T..GTCC....ACG.A     CA..T..ACCG.A.TA.GT.CTA.     C.CT.G..GTGTA.G..TT......
MERS-CoV                 GAT.TCAA.T..GA..TCC    G...C..AA.CT..T.GAT.     ...CTGG.G.G.G.TGC.TA.A..     C.A.....GTGTA.TA.A..AA...
HCoV-229E                -------------------    --------------------     -----------.G.T.ACAA.A..     G..GC.T..TTGGC.A.TT.TAAAT
HCoV-OC43                TCT---------.A.CTA.    G...C..CGATT.ATTGAT.     TT.ATGGC.C....T.A.GA.AT.     ..TG.GT.GG..A.GA.TT.TA...
HCoV-NL63                -------------------    --------------------     -----------..T....AA.CT.     G.TGCTT..TT.GC.A.T..TAAGC
HCoV-HKU1
                         LP                     B2                       B3
SARS-CoV-2               CTGCGCTTCGATTGTGTGCGT  ACGTGAGTCTTGTAAAACC      ACTCTCGTGTTAAAAATCTGAA
SARS-CoV                 .....................  ........T.A........      ....G.................
MERS-CoV                 .G..TAC.A....A....T.C  GTTCAGCC.GCA.T.T..T      T.CAGGA.AG....CCC.CTCT
HCoV-229E                AACTAA..AAGC.T...TTCA  .TAGA.CAG..TATGGC..      ..CAATCATA..TGC.CA.AG.
HCoV-OC43                TGTGTA...A.C.T..C.GTA  CT..CCCCT.CTATCT.TG      ..AA.GA.A.A...CCA.CAGT
HCoV-NL63                AACT.A...A.......TTTA  GTAG..CAT.ATATC....      ..CAAGA.TA..TGC.AA.AGC
HCoV-HKU1                T.TGTA.G.A.C.T....GT.  .TT.C.CCT.C.GCTT..G      .TAG.GAACAAGTT.TA.CACC
```

Fig. S7. Sequence analysis of seven human coronaviruses (SARS-CoV-2, SARS-CoV, MERS-CoV, OC43, HKU1, NL63 and 229E) corresponding to the LAMP primers of ORF and E gene. Identical bases are shown as dots whilst dashes indicate gaps.

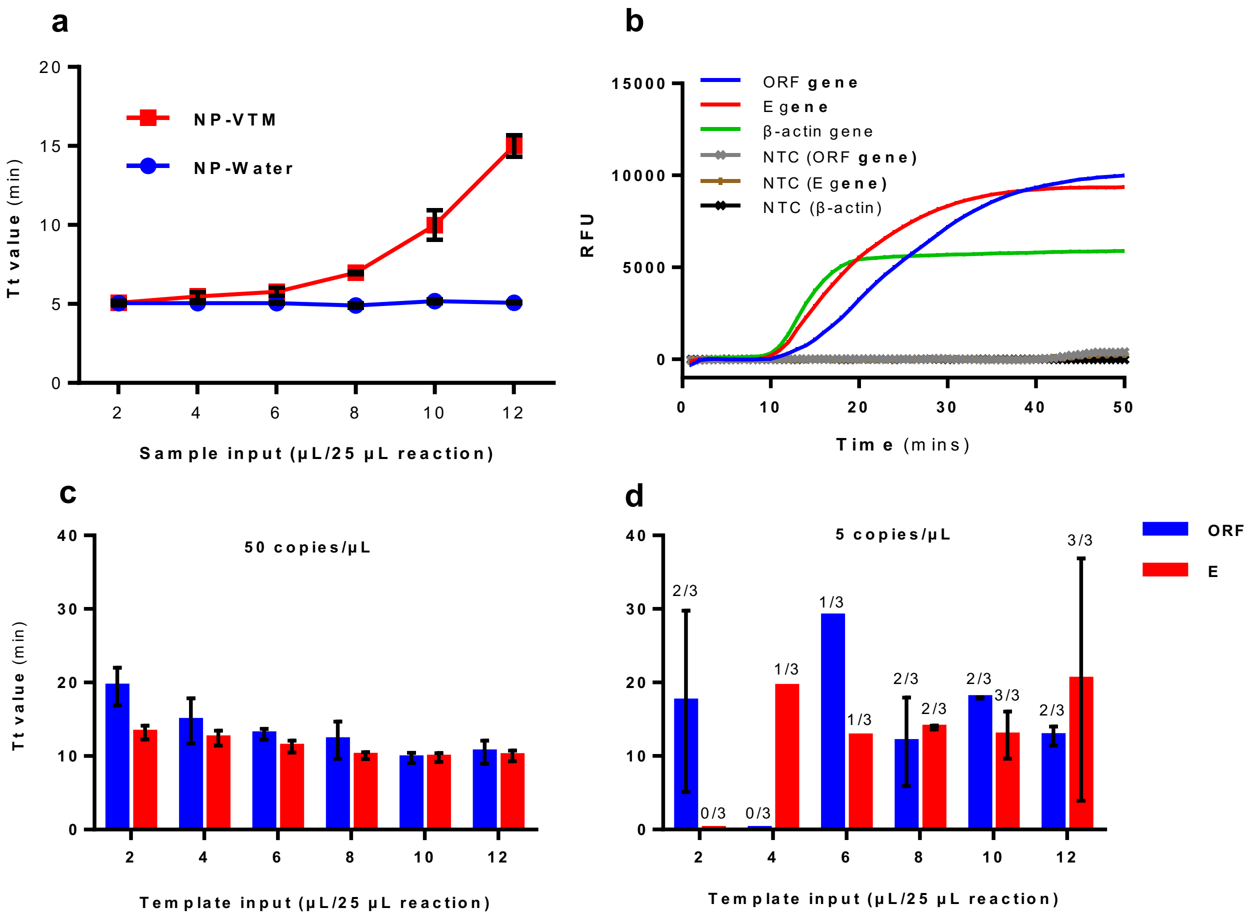

Fig. S8. Verification of the extraction-free/direct multiplex RT-LAMP assay using various inputs of simulated NP samples. (a) The influence of NP sample in different buffers (VTM and RNase-free water) on amplification efficiency of the multiplex RT-LAMP method. 10000 copies of ORF gene RNA standard were used in each 25 μL reaction. **(b)** Triplex RT-LAMP assay using nasopharyngeal swab samples (containing 200 copies of Orf and E gene RNA). (**c)** with simulated NP samples containing 50 copies of both ORF and E gene RNA standard/μL. (**d)** with simulated NP samples containing 5 copies of both ORF and E gene RNA standard/μL. 2-12 μL simulated NP samples were directed used in the multiplex RT-LAMP. The Tt means with SD are shown. The numbers above the histogram show the positive reactions and the total replicates. In panel **c**, all three replicates with different sample inputs generated positive result. NTC: no template control.

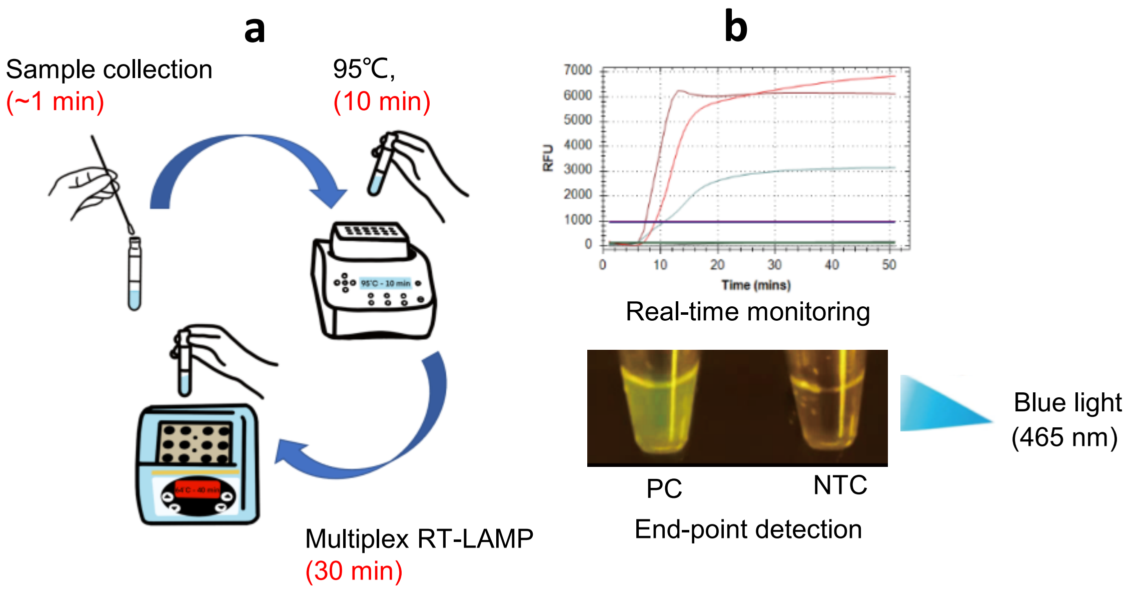

Fig. S9. The workflow of RNA extraction-free/direct multiplex RT-LAMP using nasopharyngeal swab samples. **(a)** Nasopharyngeal swab (NP) sample collection, followed by inactivation by heating (95℃, 10 min) and multiplex RT-LAMP amplification. **(b)** Results by the real-time monitoring in a real-time PCR machine and/or visual detection in 465 nm blue light. (PC is positive control showing a color change from NTC, negative control).

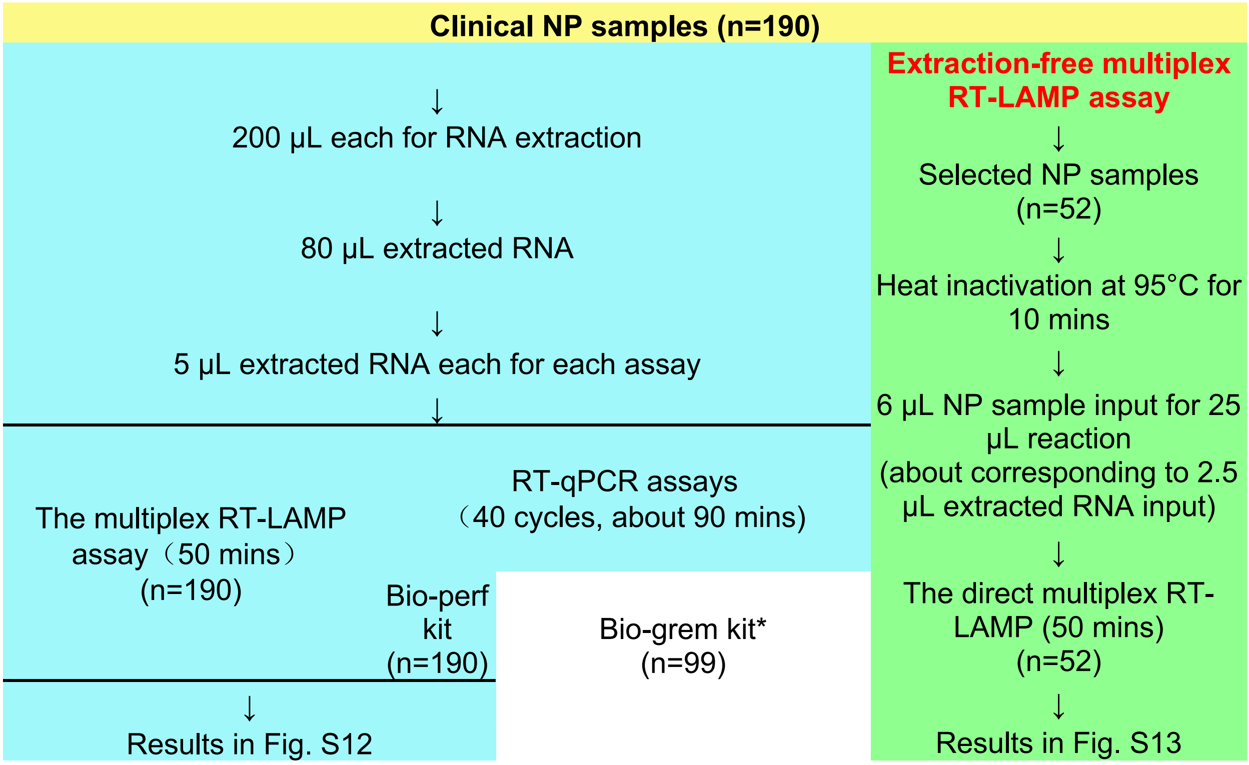

Fig. S10. Clinical validation strategy for the multiplex RT-LAMP assay using extracted RNA from NP samples and direct NP samples. * Because only 99 samples were tested by this kit, the results are shown in Supporting Fig. S11 and were not used in the formal analyses.

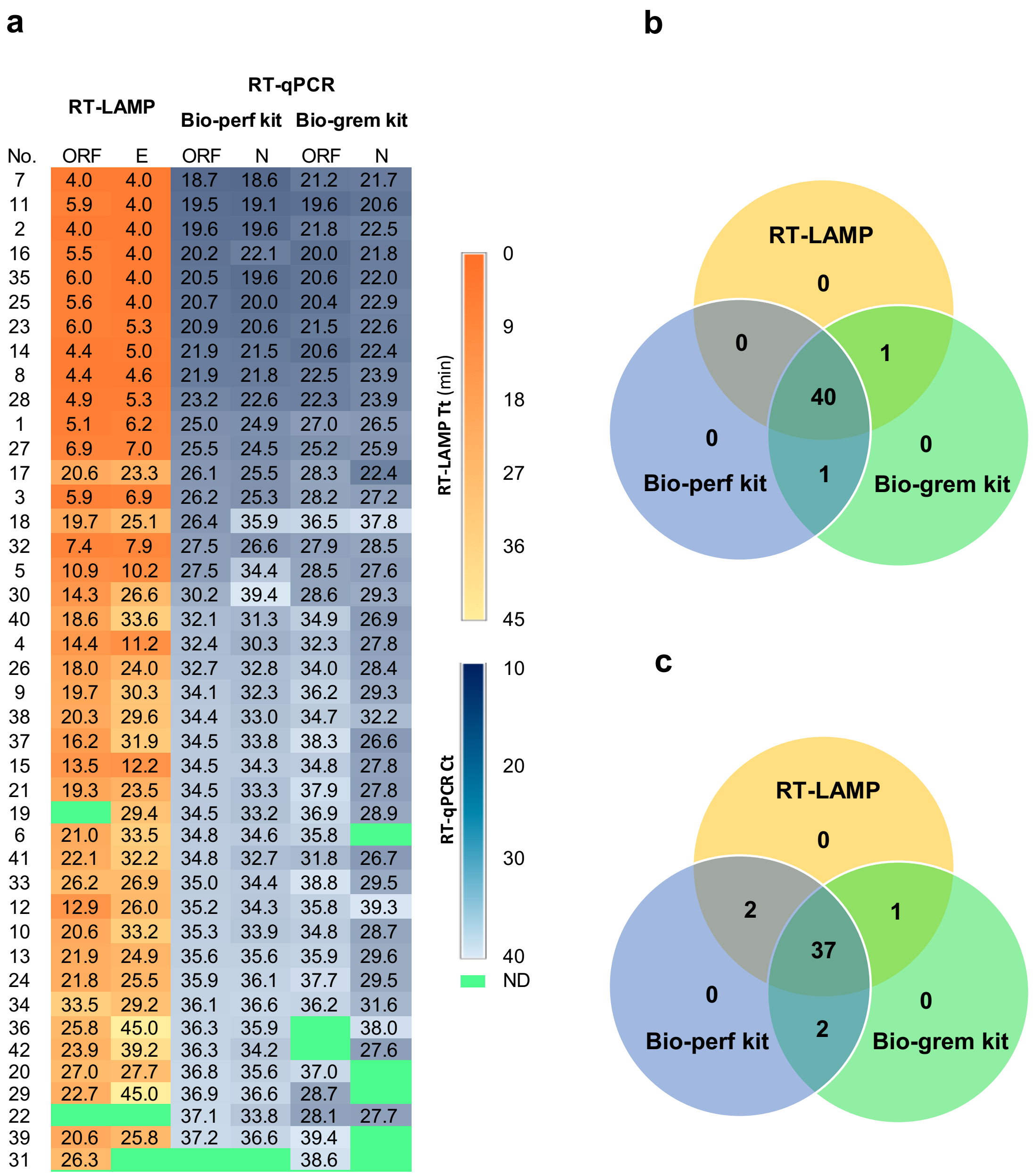

Fig. S11. Comparison of the performance of the multiplex SARS-CoV-2 RT-LAMP with two commercial RT-qPCR kits using 99 nasopharyngeal swab samples. (a) Heatmap of Tt values of the multiplex RT-LAMP assay (ORF and E genes) and Ct values of two kinds of RT-qPCR assays (ORF and N genes). Double-negative results by all three assays are not shown. (**b**) Numbers of SARS-CoV-2 positive NP samples detected by the three assays based on a single gene as output. (**c**) Numbers of SARS-CoV-2 ORF gene positive NP samples detected by the three assays. The total number of positive samples was 42 in this Venn diagram.

No.	RT-LAMP ORF	RT-LAMP E	RT-qPCR ORF	RT-qPCR N
91	4.8	4.5	16.2	13.4
84	5.2	4.7	16.7	12.7
87	5.5	4.7	17.1	11.6
7	4.0	4.0	18.7	18.6
81	5.6	5.0	18.9	12.0
11	5.9	4.0	19.5	19.1
89	6.0	5.1	19.5	16.8
67	5.7	5.1	19.5	17.0
82	5.9	5.4	19.5	15.7
2	4.0	4.0	19.6	19.6
80	5.9	5.7	19.8	16.3
16	5.5	4.0	20.2	22.1
79	5.9	5.6	20.3	17.0
35	6.0	4.0	20.5	19.6
70	6.4	5.8	20.6	15.5
25	5.6	4.0	20.7	20.0
23	6.0	5.3	20.9	20.6
66	6.1	5.1	21.1	18.6
88	6.7	5.8	21.6	17.8
72	6.1	6.0	21.6	18.6
14	4.4	5.0	21.9	21.5
8	4.4	4.6	21.9	21.8
45	6.5	5.9	21.9	18.1
52	6.9	6.1	22.4	20.4
55	6.8	6.1	22.7	19.4
78	6.6	6.3	22.9	18.3
51	6.6	5.2	23.2	20.0
28	4.9	5.3	23.2	22.6
83	6.8	6.2	24.1	20.4
44	7.2	6.6	24.5	23.0
63	7.2	7.0	24.8	21.9
71	6.9	6.8	24.8	21.4
1	5.1	6.2	25.0	24.9
27	6.9	7.0	25.5	24.5
73	8.3	6.9	25.6	21.2
17	20.6	23.3	26.1	25.5
85	7.6	6.9	26.1	22.8
3	5.9	6.9	26.2	25.3
18	19.7	25.1	26.4	35.9
50	7.7	7.1	26.5	24.1
58			26.5	24.1
65	7.7	7.0	27.0	23.8
32	7.4	7.9	27.5	26.6
5	10.9	10.2	27.5	34.4
77	8.0	7.7	27.6	24.3
74		11.5	27.8	25.2

No.	RT-LAMP ORF	RT-LAMP E	RT-qPCR ORF	RT-qPCR N
54	8.3	7.7	27.8	25.2
93	8.6	7.9	27.9	25.3
92	8.8	7.6	28.0	24.6
30	14.3	26.6	30.2	39.4
49	8.8	8.3	30.5	28.0
64	8.9	8.2	30.9	27.1
86	8.3	7.5	31.1	28.6
94	9.9	8.8	31.1	28.6
56	9.3	8.6	31.9	29.3
57	9.6	8.9	32.0	29.8
40	18.6	33.6	32.1	31.3
4	14.4	11.2	32.4	30.3
46	10.1	8.8	32.7	29.6
26	18.0	24.0	32.7	32.8
48	17.1	9.8	32.9	30.2
90	9.8	5.9	33.1	30.7
62	12.0	10.3	33.8	31.9
9	19.7	30.3	34.1	32.3
38	20.3	29.6	34.4	33.0
37	16.2	31.9	34.5	33.8
15	13.5	12.2	34.5	34.3
21	19.3	23.5	34.5	33.3
19		29.4	34.5	33.2
6	21.0	33.5	34.8	34.6
41	22.1	32.2	34.8	32.7
33	26.2	26.9	35.0	34.4
12	12.9	26.0	35.2	34.3
10	20.6	33.2	35.3	33.9
13	21.9	24.9	35.6	35.6
24	21.8	25.5	35.9	36.1
34	33.5	29.2	36.1	36.6
36	25.8	45.0	36.3	35.9
42	23.9	39.2	36.3	34.2
68	17.6	13.3	36.6	33.3
20	27.0	27.7	36.8	35.6
29	22.7	45.0	36.9	36.6
43	20.1	7.8	36.9	33.2
59		10.1	37.0	35.2
22			37.1	33.8
39	20.6	25.8	37.2	36.6
53		15.6	38.3	34.9
31	26.3			
76				35.8
60				36.8
69				39.2
*				

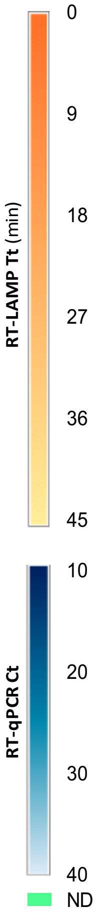

Fig. S12. Heatmap of Tt values of the multiplex RT-LAMP assay (ORF and E genes) and Ct values of a RT-qPCR assay using the Bio-perf kit (ORF and N genes) on 190 NP samples using extracted RNA, ranked by ORF gene Ct value. The NP samples were collected from 190 individuals entering Shanghai from abroad during September 2020 to June 2021. * only one of 99 negative results (samples 47, 61, 75, and 95–190) is shown. The clinical validation strategy is provided in Figure S10. ND: not detected.

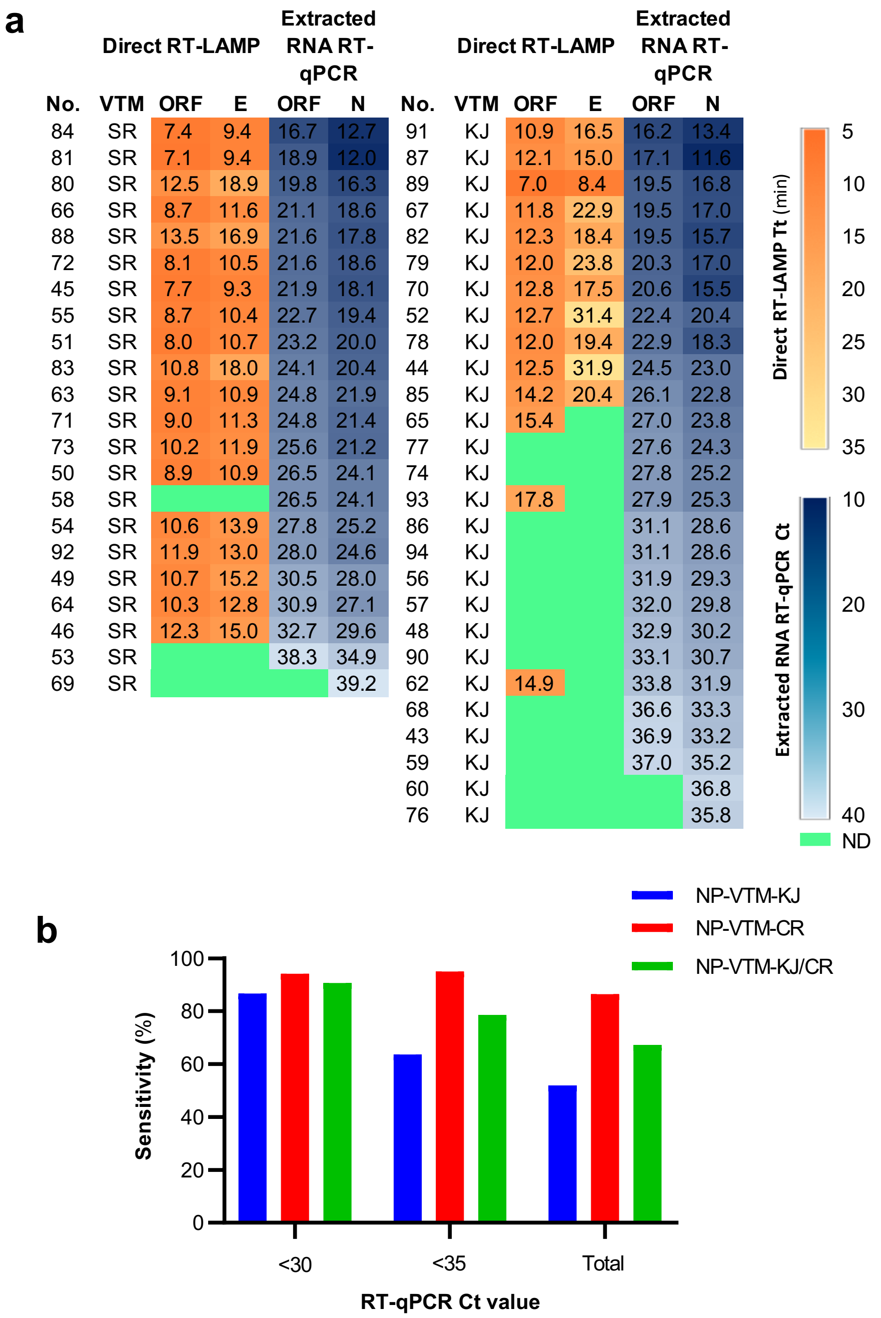

Fig. S13. Clinical validation of the RNA extraction-free/direct multiplex RT-LAMP method using nasopharyngeal swab samples. **a** heatmap of Tt values of the direct multiplex RT-LAMP assay (ORF and E genes) for 49 NP samples and Ct values of a RT-qPCR assay (ORF and N genes) using extracted RNA, ranked by ORF gene Ct value. Two kinds of commercial viral transfer media (VTM) buffers (VTM-KJ and VTM-CR) were used to collect the NP samples. The results of 3 double negative samples by two kinds of assays are not shown. **b** Sensitivity of the extraction-free multiplex RT-LAMP assay on all NP samples and those with Ct values of less 30 and 35 in the RT-qPCR assay with extracted RNA. NP: nasopharyngeal swab; VTM-KJ: viral transport media of KANGJIAN Medical; VTM-CR: viral transport media of Cienry. ND: not detected.

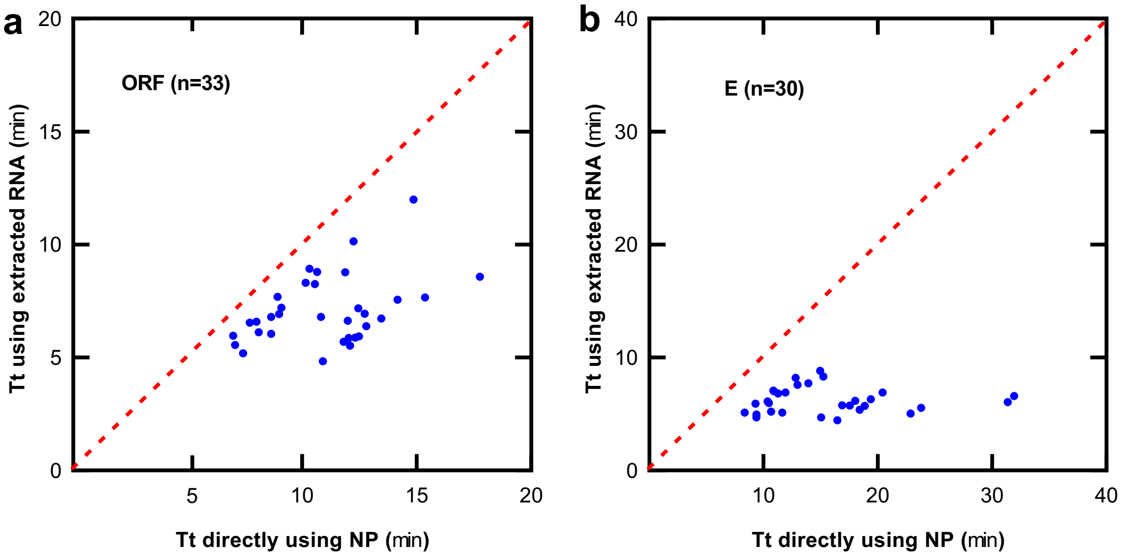

Fig. S14. Scatter plots of Tt values of ORF (a) and E (b) genes by the multiplex RT-LAMP assay with extracted RNA and direct NP samples. The Tt values of the direct multiplex RT-LAMP assay were obviously slower than the assay using extracted RNA. One reason is that the template input in the direct multiplex RT-LAMP assay was 6 μL NP sample, which corresponds to about 2.4 μL extracted RNA, half of the amount (5 μL extracted RNA) used in the standard RT-LAMP assay.

Variants/Lineages	ORF gene-LF	E gene-F2
Wuhan-Hu-1	TGGAGCACAAAACCAGTTGAAACA	ATTCGTTTCGGAAGAGACAG
Alpha(B.1.1.7)	....	....
Beta(B.1.351)	....	....
Gamma(P.1)	..............G.........	....
Delta(B.1.617.2)	....	....
Omicron(B.1.1.529)	....	.................T..
Lambda(C.37)	....	....
Mu(B.1.621)	....	....

Fig. S15 Primer sequence comparison among several major SARS-CoV-2 variants/lineages. Identical bases are shown as dots whilst dashes indicate gaps.

Supporting Tables

Table S1. The information of the primers used in this study.

Name	Sequence (5'-3')	Source
ORF-F3	TGCAACTAATAAAGCCACG	
ORF-B3	CGTCTTTCTGTATGGTAGGATT	
ORF-FIP	TCTGACTTCAGTACATCAAACGAATAAATACCTGGTGTATACGTTGTC	
ORF-BIP	GACGCGCAGGGAATGGATAATTCCACTACTTCTTCAGAGACT	G.S. Park et al. 2020
ORF-LF	TGTTTCAACTGGTTTTGTGCTCCA	
ORF-LB	TCTTGCCTGCGAAGATCTAAAAC	
ORF-LF-Probe	BHQ2-TGTTTCAACTGGTTTTGTGCTCCA-CY5	
E-F3	AGCTGATGAGTACGAACTT	
E-B3	TTCAGATTTTTAACACGAGAGT	
E-FIP	ACCACGAAAGCAAGAAAAAGAAGTATTCGTTTCGGAAGAGACAG	W.E. Huang et al. 2020
E-BIP	TTGCTAGTTACACTAGCCATCCTTAGGTTTTACAAGACTCACGT	
E-LB	CTGCGCTTCGATTGTGTGCGT	
E-LB-Probe	BHQ1-CTGCGCTTCGATTGTGTGCGT-FAM	
Actin-F3	GCGCGGCTACAGCTTCA	
Actin-B3	GGAAGAGTGCCTCAGGGC	
Actin-FIP	AAGTCCAGGGCGACGTAGCAC-CGGCCGAGCGGGAAAT	
Actin-BIP	GAGATGGCCACGGCTGCTTCC-ATTGCCAATGGTGATGACCT	This study
Actin-LB	AGAGCTACGAGCTGCCTG	
Actin-LB-Probe	BHQ1- AGAGCTACGAGCTGCCTG-HEX	
T7-Orf-F	TAATACGACTCACTATAGACACACCCTCTTTTAAGAAAGGAGCT	
Orf-R	TGTGTGGCCAACCTCTTCTGTAATT	This study
T7-E-F	TAATACGACTCACTATAGGCCTGAAGAACATGTCCAAATTCACA	
E-R	GCTCTTCAACGGTAATAGTACCGTT	
aB3-WT	Adaptor-CGTCTTTCTGTATGGTAGGAT**T**	
aB3-MutA	Adaptor-CGTCTTTCTGTATGGTAGGAT**A**	
aB3-MutC	Adaptor-CGTCTTTCTGTATGGTAGGAT**C**	This study
aB3-MutG	Adaptor-CGTCTTTCTGTATGGTAGGAT**G**	
F3	TGCAACTAATAAAGCCACG	
adaptor	TCAAACAAGCAACGAGAGTC	

Note: The sequences underlined are T7 site.

Table S2. SARS-CoV-2 variants/lineages identified from available sequencing data (53 clinical samples).

Sample No.	Variants name	Sample date
1	B.1.617.2	2021.6.19
2	B	2021.6.23
3	AY.3	2021.6.19
4	B.1.1.7	2021.5.25
5	B.1.525	2021.5.20
6	B.1.1.7	2021.5.22
7	B.1.1.523	2021.5.20
8	B.1.1.7	2021.5.19
9	B.1.1.7	2021.6.3
10	B.1.1.7	2021.6.3
11	B.1.1.7	2021.1.17
12	B.1.2	2021.1.26
14	B.1.575	2021.1.25
15	B.1.2	2021.1.2
16	B.1	2021.2.2
17	B.1.1	2021.2.14
19	B.1.1.7	2021.2.5
20	B.1.1	2021.5.4
21	B.1.1.7	2021.6.22
22	B.1	2021.6.14
23	AY.3	2021.6.14
24	B.1.1	2021.6.9
25	B.1.1.28	2021.6.12
26	B.1	2021.6.19
27	B.1.177.73	2021.1.21
28	B.1.1.7	2021.3.6
29	B.1	2021.3.22
30	B.1.1.7	2021.3.22
31	B.1.214.2	2021.3.3
32	B.1	2021.4.14
34	B.1.1	2021.3.6
35	B.1	2021.3.9
36	B.1	2021.5.4
37	B.1.596	2021.6.16
38	B.1.1.7	2021.5.4
39	B.1.1.7	2021.5.2
40	B.1.1.7	2021.5.21
41	B.1.1.7	2021.4.22
45	B.1.1	2020.12.4
46	B.1.1.129	2020.12.4
51	B.1.1.294	2020.12.4
63	B.1.177.7	2020.10.26
65	B.1.1	2020.12.15
66	A.21	2020.11.5
70	B.1.177	2020.10.28
72	B.1	2020.11.6
74	B.1	2020.12.3
78	B.1.499	2020.10.26
79	B.1.324	2020.10.6
80	A.21	2020.11.6
81	B.1	2020.11.6
85	B.1.1.372	2020.9.24
91	B.1	2020.9.13

Table S3. Feature comparison of the novel multiplex real-time LAMP with other RT-LAMP methods and the real-time qPCR.

**Features**	**LAMP**			**Real-time PCR (qPCR)**
	**Novel multiplex real-time LAMP**	**Mismatch-tolerant LAMP**	**Conventional LAMP**	
**Reaction condition**	62-65 ℃	62-65 ℃	62-65 ℃	Thermal cycle (denaturation-annealing-extension)
**Reaction time**	< 30 min	< 30min	60 min	ca. 90 min
**Specificity** (non-specific signal)	High (No)	Low (Yes)	Low (Yes)	High (No)
**Sensitivity** (copies/reaction)	1-10	1-10	> 10	1-10
**Single-tube multiplex detection**	Yes	No	No	Yes
**Tolerance to mismatches**	High	High	Low	Low
**POCT potential**	Yes	Yes	Yes	No

Supplementary References

1 Walsh, K. A.; Jordan, K.; Clyne, B.; Rohde, D.; Drummond, L.; Byrne, P.; Ahern, S.; Carty, P. G.; O'Brien, K. K.; O'Murchu, E.;et al. SARS-CoV-2 detection, viral load and infectivity over the course of an infection. *J Infect* **2020**, *81*, 357-371.
